# Evolutionary Trajectories are Contingent on Mitonuclear Interactions

**DOI:** 10.1093/molbev/msad061

**Published:** 2023-03-17

**Authors:** Damien Biot-Pelletier, Stefano Bettinazzi, Isabelle Gagnon-Arsenault, Alexandre K Dubé, Camille Bédard, Tuc H M Nguyen, Heather L Fiumera, Sophie Breton, Christian R Landry

**Affiliations:** Institut de biologie intégrative et des systèmes, Université Laval, Québec, QC, Canada; Département de biologie, Université Laval, Québec, QC, Canada; Centre de recherche en données massives (CRDM), Université Laval, Québec, QC, Canada; PROTEO, le regroupement québécois de recherche sur la fonction, l’ingénierie et les applications des protéines, Université Laval, Québec, QC, Canada; Département de sciences biologiques, Université de Montréal, Montréal, QC, Canada; Institut de biologie intégrative et des systèmes, Université Laval, Québec, QC, Canada; Département de biologie, Université Laval, Québec, QC, Canada; Centre de recherche en données massives (CRDM), Université Laval, Québec, QC, Canada; PROTEO, le regroupement québécois de recherche sur la fonction, l’ingénierie et les applications des protéines, Université Laval, Québec, QC, Canada; Département de biochimie, microbiologie et bio-informatique, Université Laval, Québec, QC, Canada; Institut de biologie intégrative et des systèmes, Université Laval, Québec, QC, Canada; Département de biologie, Université Laval, Québec, QC, Canada; Centre de recherche en données massives (CRDM), Université Laval, Québec, QC, Canada; PROTEO, le regroupement québécois de recherche sur la fonction, l’ingénierie et les applications des protéines, Université Laval, Québec, QC, Canada; Département de biochimie, microbiologie et bio-informatique, Université Laval, Québec, QC, Canada; Institut de biologie intégrative et des systèmes, Université Laval, Québec, QC, Canada; Département de biologie, Université Laval, Québec, QC, Canada; Centre de recherche en données massives (CRDM), Université Laval, Québec, QC, Canada; PROTEO, le regroupement québécois de recherche sur la fonction, l’ingénierie et les applications des protéines, Université Laval, Québec, QC, Canada; Department of Biological Sciences, Binghamton University, Binghamton, NY, USA; Department of Biological Sciences, Binghamton University, Binghamton, NY, USA; Département de sciences biologiques, Université de Montréal, Montréal, QC, Canada; Institut de biologie intégrative et des systèmes, Université Laval, Québec, QC, Canada; Département de biologie, Université Laval, Québec, QC, Canada; Centre de recherche en données massives (CRDM), Université Laval, Québec, QC, Canada; PROTEO, le regroupement québécois de recherche sur la fonction, l’ingénierie et les applications des protéines, Université Laval, Québec, QC, Canada; Département de biochimie, microbiologie et bio-informatique, Université Laval, Québec, QC, Canada

**Keywords:** mitonuclear evolution, mitochondria, evolutionary convergence, experimental evolution

## Abstract

Critical mitochondrial functions, including cellular respiration, rely on frequently interacting components expressed from both the mitochondrial and nuclear genomes. The fitness of eukaryotic organisms depends on a tight collaboration between both genomes. In the face of an elevated rate of evolution in mtDNA, current models predict that the maintenance of mitonuclear compatibility relies on compensatory evolution of the nuclear genome. Mitonuclear interactions would therefore exert an influence on evolutionary trajectories. One prediction from this model is that the same nuclear genome evolving with different mitochondrial haplotypes would follow distinct molecular paths toward higher fitness. To test this prediction, we submitted 1,344 populations derived from 7 mitonuclear genotypes of *Saccharomyces cerevisiae* to >300 generations of experimental evolution in conditions that either select for a mitochondrial function or do not strictly require respiration for survival. Performing high-throughput phenotyping and whole-genome sequencing on independently evolved individuals, we identified numerous examples of gene-level evolutionary convergence among populations with the same mitonuclear background. Phenotypic and genotypic data on strains derived from this evolution experiment identify the nuclear genome and the environment as the main determinants of evolutionary divergence, but also show a modulating role for the mitochondrial genome exerted both directly and via interactions with the two other components. We finally recapitulated a subset of prominent loss-of-function alleles in the ancestral backgrounds and confirmed a generalized pattern of mitonuclear-specific and highly epistatic fitness effects. Together, these results demonstrate how mitonuclear interactions can dictate evolutionary divergence of populations with identical starting nuclear genotypes.

## Introduction

According to the endosymbiotic hypothesis, mitochondria derive from an ancestral bacterial symbiont that gradually specialized in its current cellular functions (Dyall et al. 2004; Fritsch et al. 2014). Although most mitochondrial proteins are encoded in the nucleus, the vestigial genome of the organelle retains key genes thought to favor responsiveness to changing conditions (Allen 2003; Lane and Martin 2010; Lane 2011). Gene products encoded in the two genomes are thus required for mitochondrial function. The mitochondrion is now the site of cellular respiration and, as such, plays a central role in energy production (Malina et al. 2018). Other important metabolic processes involve the mitochondrion, including the biosynthesis of heme (Ferreira 1995), iron–sulfur clusters (Read et al. 2021), and steroids (Jordá and Puig 2020). Mitochondria are also involved in nutrient sensing, the regulation of redox homeostasis, and several signaling pathways (Malina et al. 2018).

Components encoded in the nuclear and mitochondrial genomes interact extensively, sometimes encountered together as part of mitochondrial protein complexes (Malina et al. 2018). Cytochrome *c* oxidase (Cooper et al. 1991) and the cytochrome *bc1* complex (Hunte et al. 2003), components of the respiratory electron transport chain, are assembled from proteins encoded by both genomes. In yeast, the mitogenome encodes for mitochondrial ribosomal RNAs and one ribosomal polypeptide, whereas most protein components are found in the nuclear genome (Desai et al. 2017). In humans, the nucleus-encoded protein LRPPRC is required for cytochrome *c* maturation via interaction with mitochondrial mRNAs, and mutations that alter this function are associated with severe disease (Xu et al. 2012). These examples illustrate the critical importance of mitonuclear interactions and how their disruption may severely reduce fitness.

Yet, because they are distinct and segregated to separate cell compartments, molecular evolution in the nuclear and mitochondrial genomes is partially uncoupled. Mutation rate tends to be higher in mtDNA (Lynch et al. 2008; Fritsch et al. 2014), especially in organisms with uniparental inheritance and lacking a dedicated mitochondrial mismatch repair system (Mason and Lightowlers 2003; Havird and Sloan 2016). In many organisms, uniparental inheritance also leads to smaller effective population size and the virtual lack of recombination (Lynch et al. 2006; Hagström et al. 2014). More generally, mitochondria evolve in a regulated microenvironment—the cytoplasm with its nuclear supply of mitochondrial proteins—that differs from the extracellular milieu affecting the entire cell. Illustrating this peculiar aspect of mitochondrial evolution is the suppressivity of *ρ*^−^ mitogenomes in yeast. In these cells, despite a strongly deleterious effect on organismal fitness, copies of the mitochondrial genome deleted at loci essential for respiration may outcompete healthy copies thanks to their higher replication origin density (de Zamaroczy et al. 1981; Mangin et al. 1983; Bernardi 2005). Furthermore, deletion screens in yeasts have also repeatedly identified signals of frequent essentiality switching among genes involved in mitochondrial functions, both across (Kim et al. 2010) and within species (Caudal et al. 2022; Chen et al. 2022), suggesting enhanced evolvability at these loci. In summary, because of unique characteristics and selection pressures, the rate of evolution tends to be higher in the mitochondrial genome than in the nuclear genome, with a critical influence on genetic divergence, speciation, reproductive strategies, and morphology (Hill 2015).

A model has emerged in which rapid and essentially nonadaptive changes to the mitochondrial genome exert a constant selective pressure on the nuclear genome to accumulate compensatory mutations (Dowling 2014). Rapid changes in mtDNA would drive the nucleus toward increasing divergence, the evolution of Dobzhansky–Muller incompatibilities favoring speciation via postzygotic reproductive isolation (Freel et al. 2015). Such incompatibilities are expected to arise following hybridization between allopatric populations, whose mitonuclear genotypes have evolved independently under different environmental conditions. As extensively described in the copepod *Tigriopus californicus*, hybrids may display fitness loss and reproductive incompatibility when initially separated populations come into contact, generating mismatched mitonuclear genotypes (Burton and Barreto 2012; Barreto et al. 2018; Burton 2022). A growing body of evidence accumulates in favor of this model. Early studies showed that species divergence is associated with a loss of mitonuclear compatibility (Niki et al. 1989; Kenyon and Moraes 1997; Osuský et al. 1997; Dey et al. 2000; McKenzie and Trounce 2000; Špírek et al. 2000; Yamaoka et al. 2000). More recent reports provide examples of reproductive isolation and hybrid fragility associated with mitonuclear incompatibilities (Lee et al. 2008; Chou et al. 2010; Paliwal et al. 2014; Ma et al. 2016; Haddad et al. 2018). The power of mitochondrial markers and genome structures as phylogenetic predictors further supports a driving role for the evolution of mtDNA in divergence and speciation (Robba et al. 2006; Hendrich et al. 2010; Wolters et al. 2015).

Current understanding about the cell and molecular biology of the mitochondrion owes much to methods and approaches pioneered in the yeast *Saccharomyces cerevisiae* (Rutter and Hughes 2015). As a facultative anaerobe, this yeast can be explicitly selected for respiratory function, via the use of fermentable and nonfermentable carbon sources. This facilitates cytoduction, in which mitochondrial genomes are exchanged between individuals of the same or different species, generating mitonuclear hybrids (Zakharov and Yarovoy 1977; Barrientos et al. 1998). Cytoduction and related methods have been applied to the study of evolutionary questions in mitochondrial biology, for example, investigating the compatibility of mitochondrial genomes between closely related species. Coherent with a role for mitochondria in defining the interspecific barrier, these studies showed a rapid decline in the efficiency of oxidative phosphorylation as species divergence increases between the nuclear and mitochondrial parents of mitonuclear hybrids (Kenyon and Moraes 1997; Barrientos et al. 1998; Dey et al. 2000; McKenzie and Trounce 2000; Yamaoka et al. 2000; Lee et al. 2008; Ma et al. 2016). Cytoduction has also helped identify key genes and molecular functions involved in the mitonuclear incompatibilities that arise between yeast species (Spirek et al. 2015). Collections of yeast mitonuclear hybrids have recently helped explore the genetic space of mitonuclear interactions, revealing the impact of mitonuclear interactions on fitness, and the complex third-order relationships that exist between environmental conditions, the nuclear genome, and the mitochondrial genome (Paliwal et al. 2014; Vijayraghavan et al. 2019; Nguyen et al. 2020).

Standing models thus predict that mitonuclear interactions must influence the path of evolution. Available evidence from wild individuals and populations supports this model, yet direct experimental testing of this hypothesis in naturally evolving populations is challenging. Experimental evolution in microbes is ideally suited to address this problem, because it can be replicated, enabling the observation of repeatable patterns among identically but independently evolved individuals and populations. Indeed, one of the fundamental questions that underlie much of the experimental evolution literature concerns the predictability of evolution. Given identical genetic and environmental circumstances, will independently evolving populations take the same evolutionary path? Will phenotypes be identical, will the same alleles be selected, or at least will the same genes and functional systems be affected? Repeatable outcomes have been reported in terms of both phenotype and genotype (Wichman et al. 1999; Cooper et al. 2003; Mcdonald et al. 2009; Herron and Doebeli 2013; Lang et al. 2013). Available evidence does not decisively argue for predictability (Bull and Molineux 2008; Łuksza and Lässig 2014), yet the controlled context of experimental evolution seems to lead to evolutionary convergence at the functional complex and gene levels (Tenaillon et al. 2012). Another important advantage of experimental evolution in microbes is the conservation of evolving populations at different steps of the evolutionary process. Evolved individuals can thus be compared experimentally to their ancestors. Mutations selected by evolution can be reconstituted in ancestral backgrounds and assessed for their impact on fitness using marker-assisted competition assays (Breslow et al. 2008; Lang et al. 2013), allowing the precise dissection of the genetic architecture of evolved mutants. Building on these methodological foundations, experimental evolution studies have demonstrated that the path to adaptation is contingent on the genetic makeup of the evolving population and the characteristics of its environment (Blount et al. 2008; Meyer et al. 2012). Therefore, the analysis of evolutionary convergence in replicated experimental evolution setups appears as a means to assess the impact of specific environmental and genetic factors, such as mitonuclear interactions, on evolutionary trajectories and outcomes.

In this study, we leverage a collection of mitonuclear hybrids from *S. cerevisiae* (Paliwal et al. 2014; Wolters et al. 2018; Nguyen et al. 2020) previously reported to display mitonuclear interactions in a wide array of conditions (Nguyen et al. 2020). To investigate the evolutionary impact of these interactions, we identified example strains from the collection that showed reduced fitness upon mitochondrial replacement. We performed experimental evolution on 1,344 replicate populations of the identified strains, varying the intensity of selection on mitochondrial function in an experiment spanning >300 generations. Evolving mitonuclear hybrids provided accelerated mimics of the evolutionary dynamics encountered in organisms with rapidly diverging mitogenomes. The level of replication provided us with a wealth of identically yet independently evolved individuals. We predicted that high-throughput growth assays and whole-genome sequencing on these individuals would allow us to detect patterns of evolution influenced by mitonuclear interactions, at the phenotypic or genotypic levels.

## Results

### Mitonuclear Interactions in Yeast Cybrids

**Fig. 1.**
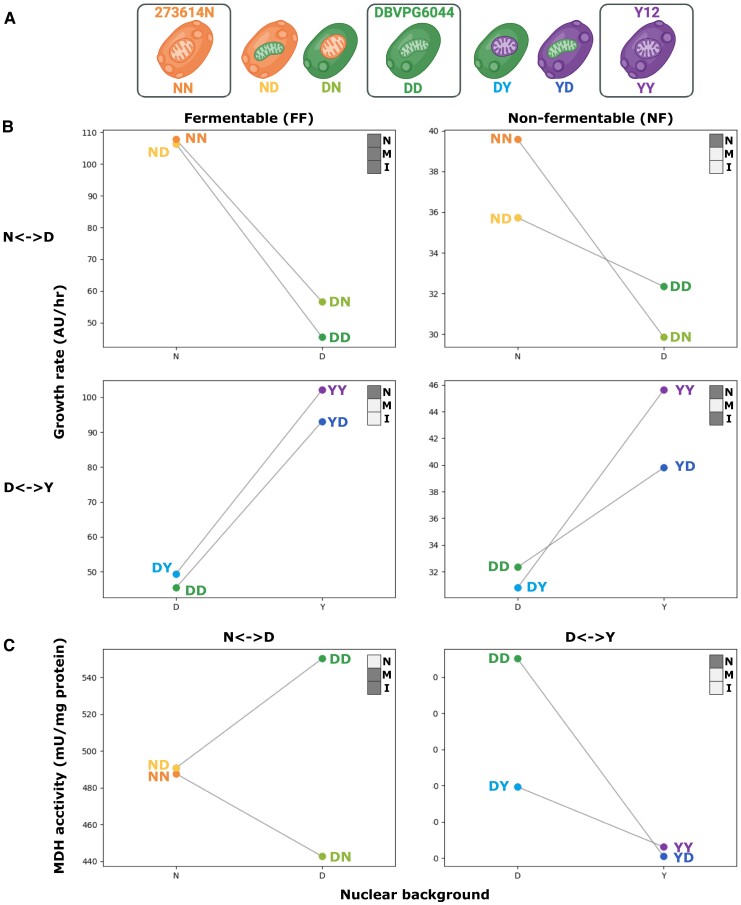
Mitonuclear interactions in founding strains of experimental evolution. (*A*) Yeast strains submitted to experimental evolution were derived from strains 273614*N* (NN), DBVPG6044 (DD), and Y12 (YY). Crosses of 273614*N* with DBVPG6044 (ND; DN) and Y12 with DBVPG6044 (DY; YD) yielded two cybrids each, identified according to their nuclear (left) and mitochondrial (right) backgrounds. We thus evolved seven ancestral mitonuclear backgrounds (panel created with BioRender.com). (*B*) Growth rate recorded for all seven strains in fermentable (FF) and nonfermentable (NF) media is reported as interaction plots for crosses of NN with DD (N < −>D) and DD with YY (D < −>Y). In each panel, the upper right corner insets indicate if nuclear (*N*) and mitochondrial (*M*) backgrounds, as well as mitonuclear interactions (*I*), have a significant effect on growth rate (two-way ANOVA *P* < 0.05). Plots report the mean values of 4 biological replicates, estimated from 8 to 42 technical replicates, for an average of 27 per point. (*C*) Malate dehydrogenase (MDH) activity also appears affected by nuclear and mitochondrial background or their interaction. Plots report the mean values of three or four biological replicates.

Our founding intent was to study the influence of mitonuclear interactions on the path of evolution. We therefore screened a collection of mitonuclear hybrids of *S. cerevisiae* for evidence of such mitonuclear interactions. This collection contains 225 cybrid strains of yeast generated by cytoduction in 4 replicates and representing all possible combinations of nuclear and mitochondrial genomes from 15 strains of *S. cerevisiae* (Nguyen et al. 2020). Though widespread mitonuclear interactions were previously found within the collection, we screened it for additional evidence, recording growth rate for all strains as a proxy for fitness in a specific condition—liquid medium with glycerol as the sole carbon source—that requires mitochondrial activity to sustain facultative anaerobic yeast. As observed previously (Paliwal et al. 2014; Wolters et al. 2018; Nguyen et al. 2020), the screen showed the predominant effect of the nuclear genome on fitness but revealed a modulating influence from the mitochondrial genome (supplementary fig. S1, Supplementary Material online). Some pairs of nuclear and mitochondrial parents displayed a depression in fitness upon cytoduction, potentially indicating mismatched nuclear and mitochondrial genomes. Prominently, mitonuclear hybrids formed between strain DBVPG6044 (D) and strains 273614N (N) and Y12 (Y) were submitted to further scrutiny to formally test the phenotypic influence of the nuclear and mitonuclear genomes and their interactions (fig. 1, supplementary fig. S2, Supplementary Material online). Following a two-letter nomenclature, indicating the nuclear background first and the mitochondrial background second, genotypes of interest were NN, ND, DN, DD, DY, YD, and YY (fig. 1*A*). These genotypes can be regarded as two sets of mitonuclear swaps: the N < −>D set consisting of genotypes NN, ND, DN, and DD and a D < −>Y swap consisting of DD, DY, YD, and YY. We estimated fitness of the strains by recording their growth rate and carrying capacity in two environments: fermentable (FF) and nonfermentable (NF) media, containing glucose and glycerol as the sole carbon source, respectively. For the two sets of mitonuclear swaps, we drew interaction plots and performed two-way ANOVAs to test the influence of both genomes and their interactions on fitness (fig. 1*B*, supplementary fig. S2, Supplementary Material online). Consistent with previous observations, the nuclear genome appeared as the main determinant of fitness, significantly affecting growth in all conditions and for all growth parameters, with the mitochondrial background playing an occasional role. Notably, the growth was significantly influenced by mitonuclear interactions in both sets. We also assayed the activity of a panel of enzymes associated with the mitochondrion in whole cell lysates of the same strains grown in nonfermentable medium (fig. 1*C*, supplementary fig. S3, Supplementary Material online). Independent effects of nuclear and mitochondrial genomes and interacting mitonuclear effects varied depending on the enzyme assays and strain set. Significant effects for mitonuclear interactions were detected for malate dehydrogenase (N < −>D) (fig. 1*C*), citrate synthase (N < −>D), and ATPase (D < −>Y) (supplementary fig. S3, Supplementary Material online). We thus accumulated a body of evidence demonstrating mitonuclear interactions by showing that interrupting coevolved mitonuclear genotypes alters fitness in strains N, D, Y, and their cybrid derivatives. This enabled further studies on the evolutionary influence of mitonuclear interactions in yeast.

### Yeast Mitonuclear Hybrids Submitted to Experimental Evolution Undergo Fitness Changes Influenced by Mitonuclear Interactions

**Fig. 2.**
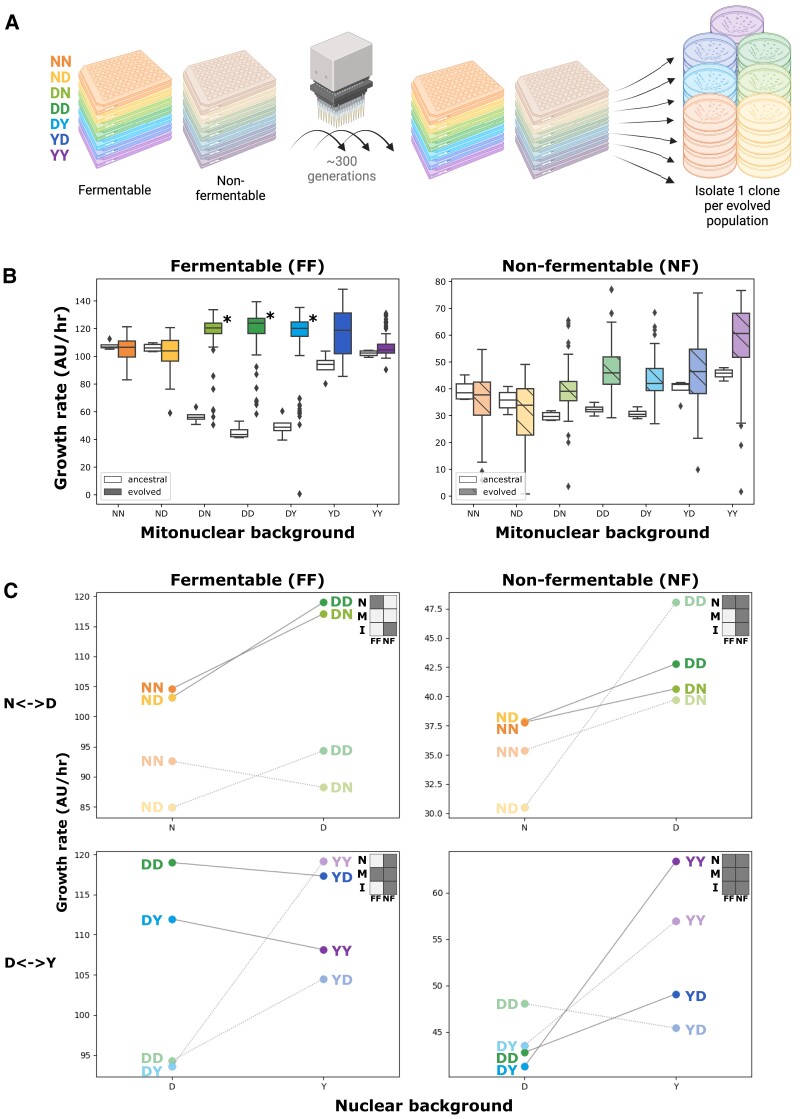
Experimental evolution in mitonuclear hybrids leads to changes in fitness in a mitonuclear background-dependent manner. (*A*) Each of the 7 founding mitonuclear backgrounds was evolved in fermentable and nonfermentable media, in 96 replicates. Evolving populations were propagated for approximately 300 generations by daily robot-assisted serial passage. At the end of the experiment, a single independently evolved individual was isolated from each population (panel created with BioRender.com). (*B*) Evolved individuals were assessed for growth rate in fermentable (left) and nonfermentable (right) media. Two-way ANOVAs indicate significant effects on growth rate for mitonuclear background and evolution. Asterisks indicate growth rate significantly above ancestral levels (Tukey HSD *P* < 0.05). (*C*) Interaction plots and two-way ANOVAs (upper right insets) indicate significant effects (*P* < 0.05) for nuclear background (*N*), mitochondrial background (*M*), or mitonuclear interactions (*I*) on growth rate in fermentable (left) and nonfermentable (right) media for strains of both crosses evolved in fermentable (dark hues, FF in inset) and nonfermentable (light hues, NF in inset) environments. Plots report the mean value for 37–91 strains, for an average of 80 per point.

We evolved 96 populations for each of the 7 mitonuclear backgrounds derived from strains N, D, and Y. This experiment was performed in conditions that require or do not require respiration using nonfermentable and fermentable carbon sources, respectively. We cultured 1,344 independently evolving populations by daily serial passage for 58 days, amounting to approximately 300 generations (fig. 2*A*). Frozen stocks were prepared at the beginning and end of the experiment, as well as every seventh passage. A subset of 230 populations went extinct or were terminated because of bacterial contamination, whereas 1,114 populations survived to the end. A single individual was isolated by streaking for single colonies from stocks of each of the surviving populations.

We next measured the extent of adaptation in evolved individuals by recording growth on both fermentable and nonfermentable media, in an automated and highly replicated manner (supplementary fig. S4, Supplementary Material online). As expected, the evolved isolates generally displayed median fitness above ancestral values, but the magnitude of the gains was dependent on the fitness of the founding strains (fig. 2*B*). Best illustrating this trend are strains carrying the D nuclear genome for which comparingly low initial fitness in fermentable media represented potential for large gains (fig. 2*B*). In comparison, mean fitness gains in fermentable medium are modest if not null in backgrounds carrying the Y and N nuclei. Interestingly, a similar endpoint fitness, close to the value of the fittest ancestors (NN, YY), is reached across backgrounds in fermentable medium suggesting that evolved clones are, on average, approaching fitness optimum in this environment. Evolution in nonfermentable medium also generally led to higher median fitness. Here also, the N nuclear genome is associated with the lowest median fitness among evolved individuals. For example, in fermentable medium, NN and ND individuals evolved in nonfermentable conditions generally appear less fit than their ancestors. This further indicates that, in fermentable medium, these strains are close to fitness optimum, where most mutations are expected to be deleterious, including those that are adaptive in other environments. Of note, in nonfermentable medium, the fittest of the ancestral backgrounds (YY) also displays one of the largest median gains in fitness, suggesting individuals evolved in this environment are still largely far from fitness optimum.

All evolved individuals were assayed for growth in fermentable and nonfermentable media. Gains achieved through evolution in one carbon source often translated into gains in the other. Indeed, in most mitonuclear backgrounds, individuals evolved in fermentable and nonfermentable medium display similar fitness in both carbon sources (supplementary fig. S4, Supplementary Material online). Strikingly, in nonfermentable conditions, strains evolved in this medium are frequently matched or outperformed by those evolved in fermentable medium (supplementary fig. S4*B*and*D*, Supplementary Material online), as they likely spent a portion of each evolution cycle respiring in the stationary phase. Similarly, in fermentable medium, although nonfermentable-evolved individuals tend be less fit than their fermentable-evolved counterparts, many still demonstrate significant fitness gains over ancestral levels (supplementary fig. S4*A*and*C*, Supplementary Material online). These observations suggest that much of the gain in fitness is related to aspects common to both environments, either in terms of media composition or culture conditions.

We used the fitness of evolved individuals to test the effect of mitonuclear interactions on evolutionary outcomes. We drew interaction plots and performed two-way ANOVAs, revealing patterns of relative fitness that bore both resemblances and differences with those of ancestral strains (fig. 2*C*, supplementary fig. S6, Supplementary Material online). Nuclear effects on fitness are most frequent in both ancestral and evolved strains. However, evidence for the influence of the mitochondrial background and mitonuclear interactions is more frequent in fitness data of the evolved strains. These results show an influence for mitonuclear interactions on the outcomes of experimental evolution at the phenotype level.

### Sequencing of Evolved Genomes Reveals Patterns of Evolutionary Convergence

**Fig. 3.**
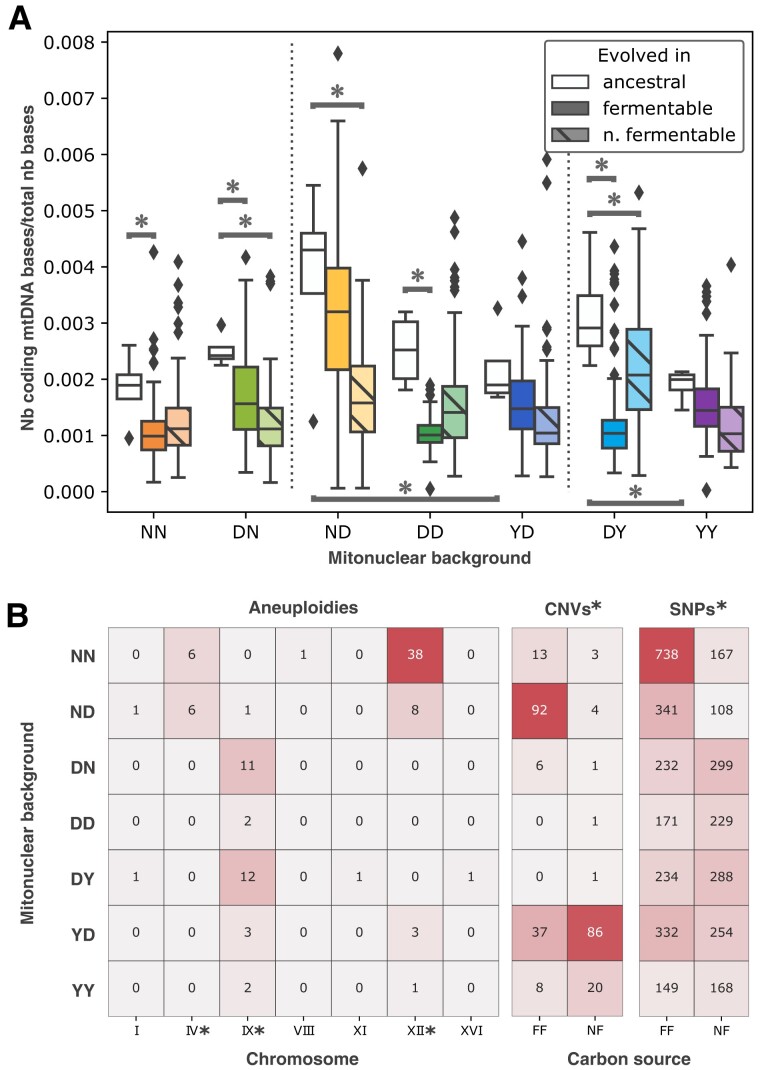
Whole-genome resequencing reveals mitonuclear background- and carbon source-specific genomic changes in experimental evolution isolates. (*A*) Fraction of sequenced bases mapped to coding regions of the mitochondrial genome was used as an indicator of genome-scale alterations to mtDNA, revealing impacts of mitonuclear background and evolution. Vertical dotted lines segregate strains based on mitochondrial background. Two-way ANOVAs comparing mtDNA fraction among strains bearing the same mitochondrial background reveal a significant effect for the nuclear background and evolution regimen (*P* < 0.05). Connectors marked by asterisks indicate significant differences in mtDNA fraction between ancestral strains bearing the same mitochondrial genome or between ancestral strains and their offsprings (Tukey HSD *P* < 0.05). (*B*) Changes in chromosome copy numbers (aneuploidies) and smaller, gene-scale copy number changes (CNVs) were detected using sequencing depth of coverage. Many single-nucleotide changes (SNVs) were also called. Asterisks indicate uneven distribution between mitonuclear backgrounds and carbon sources (*χ*^2^ test *P* < 0.05).

To probe the molecular mechanisms involved in the adaptation of evolved isolates, we sequenced each of their genomes, along with that of their ancestors, aligning to published sequences of ancestral nuclear and mitochondrial backgrounds. Our partially redundant data set comprised 1,505 nonempty sequencing libraries and provided genomic data for 1,114 evolved isolates and 28 ancestral strains. With a 31× median depth of coverage, most libraries were suitable for the confident calling of mutations, from the whole-chromosome to single-nucleotide scales (supplementary data S1, Supplementary Material online). We first examined chromosome-scale changes, beginning with the mitochondrial genome. In contrast with published methods that rely on median depth at stably covered loci *COX1* or *COX3* (Puddu et al. 2019), and acknowledging the complex and highly branched topology of *S. cerevisiae* mtDNA (Bendich 1996, 2007), we abstained from claims about mitogenome copy number and used the fraction of sequence bases mapped to coding regions of the mitochondrial genome to probe general changes to mtDNA, including structure, copy number, and others, incurred via experimental evolution and cybridization (fig. 3*A*). Because of disparities in the sequence, structure, and quality of the reference mitochondrial genomes, we restricted comparisons to strains that share the same mitochondrial background. We observed changes in mtDNA among the ancestral mitonuclear hybrids, echoing enzyme assays (supplementary fig. S3, Supplementary Material online) hinting to changes in mitochondrial metabolism upon cytoduction. We also observed a trend toward a decrease in the fraction of coding mtDNA in evolved isolates. This change is likely not the result of widespread petite genotypes, since most isolates display fitness in nonfermentable media that is equal or superior to their nonevolved ancestors (supplementary fig. S4*B*and*D*, Supplementary Material online). Restricting the analysis to euploid individuals further indicates that the pattern of apparent reduction in mtDNA is not an artifact of aneuploidies (supplementary fig. S5, Supplementary Material online). Similarly, the extent of the contraction in mtDNA fraction is dependent on both mitonuclear background and evolution conditions. Because environmental demands on respiratory metabolism affect mtDNA copy numbers, our observations about mtDNA fraction are chiefly relevant to the fermentable conditions in which we propagated the yeast used for gDNA extraction (Galeota-Sprung et al. 2022).

We detected copy number increases for several chromosomes, prominently including chromosomes XII, IV, and IX (fig. 3*B*, supplementary fig. S7, Supplementary Material online). We detected aneuploidies at chromosome XII in 50 independently evolved strains: these changes were significantly enriched in the presence of the N nuclear genome and the matching mitochondrial genome. We observed a similar pattern for aneuploidies of chromosome IV. We most often found extra copies of chromosome IX in mitonuclear hybrid derivatives of strain D (fig. 3*B*). Along with changes in mtDNA, these repeated large-scale genomic changes suggested a pattern of convergent evolution influenced by the identity and interaction of nuclear and mitochondrial genomes.

We next identified smaller scale genetic changes. Following stringent bioinformatics pipelines, we called 272 copy number variants (CNVs), ranging in size from 184 to 1,091 bp, and 3,710 single nucleotide variants (SNVs) (supplementary data S3, Supplementary Material online). Neither CNVs nor SNVs were distributed randomly among mitonuclear backgrounds and carbon sources (*χ*^2^ test *P* < 0.05). This fact may appear most obvious observing that only nine CNV calls were made in nuclear derivatives of strain D. In stark contrast, ND derivatives evolved in fermentable medium and YD derivatives evolved in nonfermentable medium accumulated 92 and 86 CNVs, respectively (fig. 3*B*). One may note that both backgrounds derive from mitochondrial swaps. In the same way, SNVs are unevenly distributed, yet the large number of calls ensures that more than a hundred mutations were detected per mitonuclear background in each carbon source. Together, these small-scale changes provided further hints that both mitonuclear interactions and the environment inflected adaptive trajectories in our mitonuclear hybrids.

### Dissimilar Mutational Profiles Provide Evidence for Mitonuclear Specific Evolutionary Trajectories

**Fig. 4.**
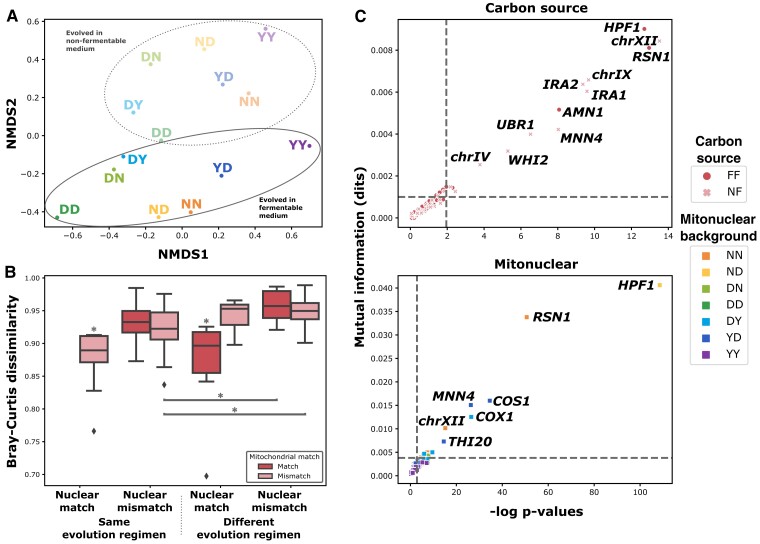
Unique evolutionary trajectories are characterized by mitonuclear and carbon source-specific patterns of convergence. (*A*) Bray–Curtis dissimilarity computed between the mutational profiles (vector of mutation counts at each mutant annotation) of all pairs of mitonuclear backgrounds evolved in each carbon source was used as input for nonparametric multidimensional scaling, revealing their relative differences. (*B*) Dissimilarity between mutational profiles is high in all conditions but modulated by a shared environment as well as common nuclear and mitochondrial backgrounds (three-way ANOVA *P* < 0.05). Boxes marked by asterisks (*) indicate evolutionary circumstances associated with significantly lower dissimilarity than all other circumstances (Tukey HSD test *P* < 0.05). Asterisk (*)-marked connectors indicate pairs of evolutionary circumstances associated with significantly different levels of dissimilarity (Tukey HSD test *P* < 0.05). (*C*) Mutant loci were considered specific to a certain carbon source (top) or mitonuclear background (bottom) if their distribution differed significantly from that of all detected mutations, taken as a whole. *χ*^2^ tests were performed on contingency tables of all mutations, classifying them as mapping or not to the locus and according to either carbon source or mitonuclear background. Mutual information was calculated from the same contingency tables. Specificity thresholds, indicated by dotted lines, were set at a *P* value of 0.05, corrected for false-discovery rate, and at two standard deviations above mean (∼95th percentile) mutual information across all loci.

From the distribution of aneuploidies and CNVs, we hypothesized that each mitonuclear background and each carbon source could be associated with at least partially distinct sets of mutations. We tested this hypothesis by calculating the Bray–Curtis dissimilarity between the sets of mutations accumulated by each background in each environment (supplementary fig. S8, Supplementary Material online). Although variable, mean dissimilarity between the mutational profiles of all pairs of evolutionary conditions is high at 0.93. To better grasp the influence of carbon sources and nuclear and mitochondrial backgrounds on mutational profiles, we used the dissimilarity matrix as input for nonparametric multidimensional scaling (NMDS, fig. 4*A*). Strikingly, this analysis reveals a broad pattern in which backgrounds evolved in the same environment cluster together along a single axis. A shared nuclear background is also associated with smaller distances and a tendency to cluster together. This analysis also suggests that the mitochondrial background more modestly influences mutational divergence. Visual examination of the dissimilarity matrix (supplementary fig. S8, Supplementary Material online) also suggested that a shared nuclear background or evolution in the same environment could be associated with reduced dissimilarity in mutational profile. We formally tested these intuitions by partitioning the dissimilarity matrix according to match or mismatch with respect to environment, nuclear background, and mitochondrial background (fig. 4*B*). We performed a three-way ANOVA on the data partitioned in this way, revealing a significant effect on dissimilarity for all three matches and their interactions. Lowest dissimilarities are associated with pairs that display a single mismatch, either at the mitochondrial or environmental levels, whereas mismatched nuclei suffice to maximize dissimilarity. Highest dissimilarity is observed when both the environment and the nucleus differ, regardless of the mitochondrion. Together, these results confirm NMDS outcomes by ascribing a major role in mutational divergence to the nuclear background and identifying the environment as more impactful than the mitochondrial background. Critically, all three aspects impact evolutionary divergence and interact to yield dissimilar mutational profiles.

We next asked if patterns of specificity were observed at the gene level and examined annotations associated with CNV and SNV calls (supplementary data S4, Supplementary Material online). Like aneuploidies, gene-level annotation indicated evolutionary convergence at several loci. Indeed, hundreds of genes are associated with more than 1 mutation among evolved clones, and 43 display 10 or more. Along with aneuploidy at chromosome XII, nuclear genes *RSN1*, *HPF1*, *MNN4*, and *AMN1* and mitochondrial gene *COX1* are the annotations associated with the strongest signals of evolutionary convergence, with tens to more than a hundred calls per locus mapping to these areas of the genome. Examining mutation counts associated with these loci in each mitonuclear background and carbon source (e.g., supplementary fig. S9, Supplementary Material online), we detected several examples of potential specificity.

Our survey of genetic changes in evolved individuals, at all scales examined, thus provided evidence for evolutionary convergence within a given mitonuclear background and strongly suggested that adaptive changes followed a pattern of specificity to both mitonuclear background and environmental conditions. To test this hypothesis, for each annotation, we performed *χ*^2^ tests of independence on contingency tables partitioning all mutations according to their association with the annotation and according to either mitonuclear background or carbon source. We also computed mutual information from the same contingency tables. Both a significant *P* value from this test and significantly above average mutual information were required to identify specific annotations (fig. 4*C*). Similar computations were performed, partitioning mutation counts according to nuclear and mitochondrial backgrounds alone or conjunctions of background with carbon source (supplementary fig. S10, Supplementary Material online). These analyses revealed areas of the yeast genome biased toward de novo mutation in a mitonuclear background- and carbon source-specific manner. For example, out of 50 aneuploidies detected at chromosome XII, 38 were found in background NN, and they appear to have been selected almost exclusively in nonfermentable medium. Similarly, 94% of the 88 CNVs at gene *HPF1* were selected in background ND evolving in fermentable medium (supplementary fig. S9, Supplementary Material online). From this data and our earlier analysis of mutational dissimilarity, we concluded that interactions of the environment with both nuclear and mitochondrial backgrounds inflected evolutionary trajectories at the molecular level.

### Reconstituting Loss-of-Function Mutations in Ancestral Backgrounds Leads to Mitonuclear Background-Specific Fitness Effects

Collected evidence suggested each mitonuclear background accumulated a distinct set of mutations during adaptive evolution. Therefore, we hypothesized that the phenotypic effects of individual mutations selected during the experiment were epistatic with the mitonuclear background. We tested the statistical association between mutant annotations and available phenotype measurements and found several candidate causal loci (supplementary fig. S11, Supplementary Material online). From this list of loci, patterns of convergent evolution, and both mitonuclear and carbon source specificity, we identified a core set of annotations potentially involved in adaptation or otherwise implicated in the evolution of our mitonuclear hybrids. Mapping of mutations on these annotated features and prediction of their mutational effects revealed widespread stop codons alongside missense mutations at conserved positions (e.g., supplementary fig. S12, Supplementary Material online). We therefore hypothesized that loss-of-function at these and other loci was a widespread adaptive mechanism selected by our experiment, as observed for other experimental populations evolved in constant environments (Kvitek and Sherlock 2013).

**Fig. 5.**
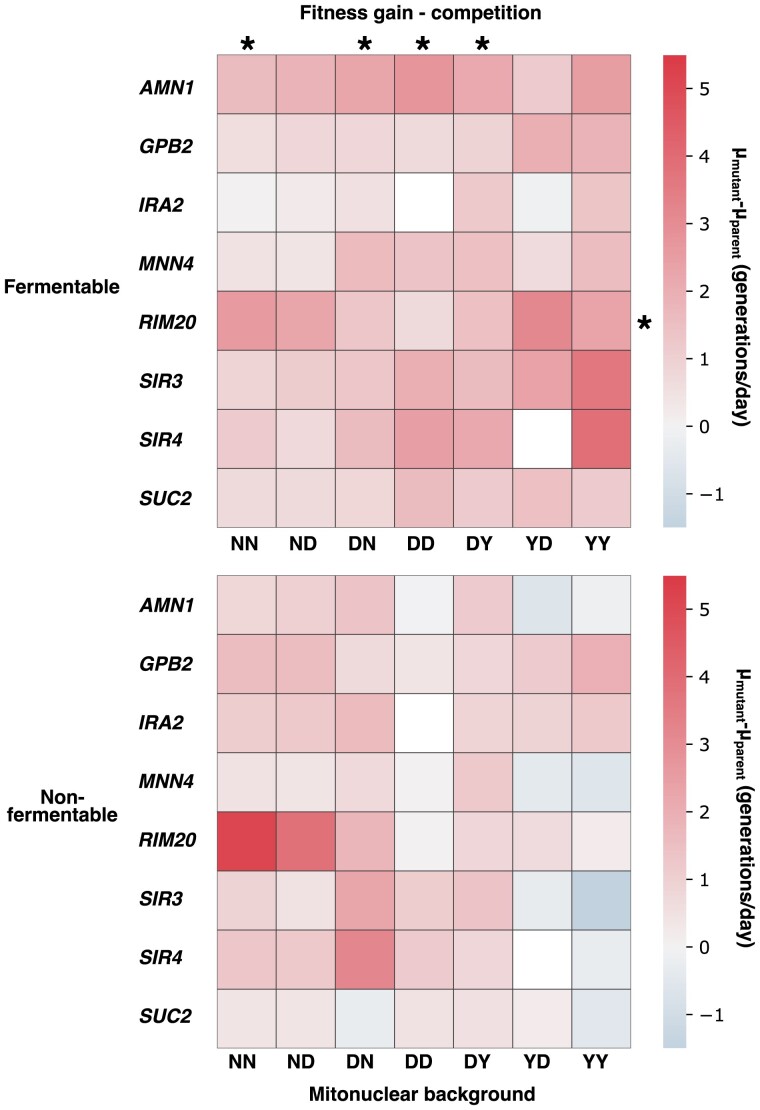
Fitness effect for loss-of-function at select loci is dependent on mitonuclear background and carbon source. Gene knockouts were performed at the loci indicated on the left in fluorescent derivatives of all seven ancestral strains used in the evolution experiment, mimicking the effect of loss-of-function alleles. These knockouts were competed against their deletion-free counterparts of the same mitonuclear genotype in fermentable (top) and nonfermentable (bottom) media. This enabled estimation of the fitness effect of loss-of-function at these loci relative to the ancestor. For all loci, effect of deletion was found to differ significantly between mitonuclear genotypes and in a manner influenced by mitonuclear interactions (two-way ANOVA *P* < 0.05). Fitness effect of each locus over mitonuclear genotypes did not correlate (Spearman rho < 0.95, *P* < 0.05 that slope of regression = 0) with frequency of mutation at the locus in each genotype, with one exception indicated by an asterisk on the right-hand side. Asterisks at the top of heatmaps indicate genotypes that display rank order correlation between locus fitness effect preference and frequency of mutation at the locus (Spearman rho > 0.7, *P* < 0.05 that slope of regression = 0).

To mimic the effect of loss-of-function alleles, we generated a replicated set of knockouts in fluorescent derivatives of each of the seven ancestral backgrounds at eight loci identified in our data set. Loci were chosen based on frequency of mutation, statistical association with changes in phenotype, technical feasibility, gene essentiality, and both mitonuclear and carbon source specificity. We competed the knockouts against their wild-type counterparts in the conditions of the evolution experiment. From these competitions, we estimated the fitness effect of loss-of-function at these loci in all mitonuclear backgrounds and both fermentable and nonfermentable media (fig. 5). Loss-of-function at these loci led in most cases to significant gains in fitness, validating their roles in adaptive evolution. The magnitude of the changes in fitness associated with each knockout differed significantly between mitonuclear backgrounds and was influenced by nuclear and mitochondrial backgrounds as well as mitonuclear interactions (two-way ANOVA *P* < 0.05, supplementary fig. S13, Supplementary Material online). Some loci even showed opposite effects between different mitonuclear backgrounds, further underlining the strongly epistatic nature of their impact on fitness. This result provided additional support for a decisive role of mitonuclear interactions in shaping and inflecting the path of adaptive evolution.

Competition assays provided additional support for evolution contingent on mitonuclear interactions. Yet, for most tested loci, differential fitness effect patterns did not match predictions derived from our analysis of evolutionary convergence (fig. 5, supplementary fig. S14, Supplementary Material online). Contrary to expectation, apart from *RIM20* knockouts growing in fermentable medium, rank correlation was poor between estimated fitness effects and mutation frequency in evolved mutants (Spearman correlation *P* on the slope > 0.05). In other words, frequent mutation at a locus does not reliably predict strong gains in fitness in knockouts of that locus. In contrast, in fermentable medium, predictions of locus preference based on mutation frequency matched fitness effect-based predictions of preference for half of our mitonuclear backgrounds (fig. 5, supplementary fig. S14, Supplementary Material online).

We explored possible explanations for the partial disconnect between convergence- and fitness effect-based predictions. The experiment was based on the simplifying assumption that complete loss-of-function is the primary mechanism underlying the fitness effect of mutation at all examined loci. Different or concurring mechanisms are likely involved and may explain some of the discrepancy between fitness data and our analysis of evolutionary convergence. Such mechanisms may include protein truncation, loss of regulatory interactions, and partial reductions in activity, to name but a few. Yet, beside the raw fitness effect of mutations in the ancestral strains, other mechanisms not related to mitonuclear interactions may account for the observed patterns of mitonuclear and carbon source specificity. Fitness of the ancestral strains (fig. 1*B*, supplementary fig. S2, Supplementary Material online) differs markedly between mitochondrial backgrounds. If the fittest backgrounds are indeed approaching fitness optimum, as hypothesized above, and given patterns of diminishing returns epistasis common in experimental evolution (Chou et al. 2011; Wiser et al. 2013; Couce and Tenaillon 2015), the magnitude of fitness gains accessible to different mitonuclear pairs likely differed, constraining evolutionary paths to adaptation. Considering that initial fitness itself is influenced by mitonuclear interactions, this effect may prove difficult to deconvolute from other similarly influenced mechanisms. However, we did identify loci uniquely or preferentially mutated in mitonuclear backgrounds that otherwise display comparable initial fitness to closely related backgrounds (supplementary fig. S9, Supplementary Material online). We also counted the number of nonsynonymous mutation calls in evolved individuals. We found that the median number of mutations detected per evolved individual is two (supplementary data S3, Supplementary Material online). Hence, in evolved individuals, loss-of-function alleles normally interact with other mutant loci, contrary to our simplified experiment, performed in the ancestral backgrounds. This aspect appears critical when considering mutational order: Selection for mutation at any given locus is contingent on the identity of mutations most likely to fix earlier during evolution (Weinreich et al. 2006). This itself may be influenced by mitonuclear interactions and illustrates the difficulty of predicting patterns of mutational specificity and evolutionary convergence based solely on fitness effects measured in the ancestral strains.

We also found that the nucleotide-level mutational profile observed in backgrounds NN and YY differed slightly but significantly from that of all other strains (supplementary fig. S15, Supplementary Material online). This mutational bias would support the hypothesis that patterns of background-dependent evolutionary convergence may, at least in part, be dictated by differential access to beneficial mutations rather than mitonuclear interactions. Yet, because the nuclear genome, by virtue of its much larger size, chiefly determines mutational bias, this hypothesis does not account for differences in mutational specificity observed between backgrounds that carry the same nuclear genome (as illustrated in supplementary fig. S9, Supplementary Material online). Moreover, fitness effects inferred from association tests in evolved mutants also correlated poorly with both convergent evolution signals and competition data (supplementary fig. S14, Supplementary Material online). Together, these results indicate that different mitonuclear backgrounds may have access to different mutational spectra, in the presence of multiple and complex epistatic networks. Mitonuclear interactions are a critical component of these epistatic networks and appear to exert a decisive impact on the path of evolutionary adaptation in *S. cerevisiae*.

## Discussion

The current model of mitonuclear evolution proposes that the mitochondrial genome, through interactions with nucleus-encoded genetic material, exerts an influence on evolutionary divergence of populations and eventually on the process of speciation. An elevated rate of evolution observed in the mitogenome can exert a pressure for compensatory evolution of the nuclear genome, leading to negative interactions between mitonuclear pairs that have evolved independently (Dowling 2014). Evidence of mitonuclear incompatibility between both closely related species (Niki et al. 1989; Kenyon and Moraes 1997; Osuský et al. 1997; Dey et al. 2000; McKenzie and Trounce 2000; Špírek et al. 2000; Yamaoka et al. 2000; Lee et al. 2008; Chou et al. 2010; Ma et al. 2016) and geographically isolated populations of the same species (Paliwal et al. 2014; Wolters et al. 2015, 2018; Barreto et al. 2018; Nguyen et al. 2020) corroborates this model. Yet, direct experimental evidence demonstrating the impact of mitonuclear interactions on evolutionary trajectories and outcomes was still lacking. We designed an experimental evolution study aimed at filling this gap. We mimicked the potential pressure exerted by the mitochondrial genome on the nuclear genome by swapping mitochondrial genotypes among yeast strains. We propagated these strains in a highly replicated manner for several hundred generations in conditions that either explicitly select for a mitochondrial function or relax the selective pressure exerted on this function. If the mitochondrial genome exerts a pressure on the nuclear genome, independent populations of the same nuclear genotypes but with distinct mitochondrial haplotypes should converge on different adaptive mutations.

### Mitonuclear Interactions and Their Impact on Evolutionary Trajectories

The outcomes of our evolution experiment and the analyses herein support our prediction that specific mitonuclear interactions led to distinct evolutionary trajectories. Our phenotypic data show the influence exerted on fitness of mitonuclear interactions in both ancestral and evolved individuals. Such effects depended on the conditions in which they were tested, the evolution regimen, and fitness proxies retained for analysis. Fitness in the evolved individuals that we isolated is on average comparable albeit not identical among the mitonuclear backgrounds under study. Genomic scrutiny in those individuals provides additional evidence for the impact of mitonuclear interactions on evolutionary outcomes. Thanks to our highly replicated data set, we were able to detect signals of evolutionary convergence within starting genotypes at the whole-chromosome and gene levels, as observed in previous evolution experiments (Tenaillon et al. 2012). This allowed us to identify features of the yeast genome specifically or preferentially targeted by selection in a mitonuclear background-specific manner. This included changes that were specific to one single mitonuclear background, to the exclusion of closely related ones. We similarly detected environment-specific patterns of selection. Furthermore, recapitulating loss-of-function alleles at convergent loci in the ancestral strains of our experiment, we demonstrated that their effect on fitness, although generally favorable, varied markedly as a function of mitonuclear background and carbon source. Taken together, this evidence therefore upholds standing models of mitonuclear evolution, whereas specific interactions between the nuclear and mitochondrial genomes lead to divergent evolutionary trajectories.

Screening our collection of mitonuclear hybrids, we observed that the starting nuclear genome exerted a larger impact on fitness than did the mitochondrial genome. Highly replicated assessment of fitness in the ancestral strains confirmed this observation but indicated a modulating influence for the mitochondrial genome and mitonuclear interactions. Further supporting this view, we found evidence that the disruption of co-evolved mitonuclear pairs via cytoduction can impact the activity of mitochondrial enzymes and is associated with changes in mtDNA content. These founding contingencies in the experimental evolution represent, on their own, strong predictors of the divergent evolutionary trajectories predicted from different mitonuclear backgrounds. Indeed, the contingent evolutionary history of an organism creates a unique ensemble of constraints on the set of alleles likely favored by selection. In other words, it potentiates specific evolutionary pathways, and examples of this are reported in the experimental evolution literature (Blount et al. 2008).

Evolved individuals displayed greater phenotypic uniformity than their unevolved ancestors. Less adapted mitonuclear backgrounds readily caught up to fitter ones. This is coherent with the rapid and reproducible gains in fitness generally observed in the early stages of experimental evolution (Wiser et al. 2013) and with patterns of diminishing returns epistasis expected in fitter individuals (Chou et al. 2011; Couce and Tenaillon 2015). Evolved individuals display phenotypic uniformity in the environment in which they evolved, but also in conditions to which they were not explicitly adapted. This could be a sign of positive pleiotropy, meaning that many of the mutations selected in one environment tend to be adaptive in the other. More prosaically, we note that the two environments differ solely by the identity of the available carbon source. Hence, we posit that both environments are more alike than different and alleles selected in either will tend to be adaptive in both by virtue of the same mechanisms. Despite this, we did observe several environment-specific mutant annotations. We also observed slightly lower fitness in fermentable medium for individuals evolved in nonfermentable medium. Therefore, environment-specific adaptive strategies still arose, which may steer the path of evolution toward more strongly distinct pathways over a longer evolutionary period.

Mitonuclear cooperation posits a complex network of genetic and physical interactions. Compatible with this framework, patterns of mitonuclear-specific evolutionary convergence and mitonuclear interactions presented in this study suggest selection affected by high-order epistasis. Recapitulating some of the selected mutations as loss-of-function alleles further argued for that view, as their effect in the ancestral backgrounds was dependent on the mitonuclear background. Furthermore, the locus preferences we could infer from the fitness of these reconstituted mutants did not match predictions from patterns of evolutionary convergence. Since we know that each evolved individual carries more than one mutation, we can reasonably posit that the effect of mutation at any locus in our mutants, and the probability for their selection during evolution, is affected by other mutations found in the same cells, especially those that arose earlier. The factor that unifies all these considerations is the founding difference in mitonuclear background.

### Future Directions

The model of mitonuclear evolution tested in this study predicts that evolutionary divergence is driven by the high rate of evolution experienced by the mitochondrial genome. In turn, the nuclear genome is believed to accumulate compensatory mutations aimed at maintaining compatibility. Data presented in this study support this model by showing the influence of specific mitonuclear interactions on the evolutionary trajectories and range of selected mutations observed in adapting mitonuclear hybrids. We also showed evidence of mitonuclear incompatibility and metabolic disruption in the mitonuclear hybrid ancestors of the experiment. In addition, we provided evidence for the resolution of the incompatibilities. However, this study shows no evidence about the site of compensatory evolution in either the nuclear or mitochondrial genomes. Strains evolved during our experiment may yet enable such demonstrations. Using cytoduction, we may generate novel mitonuclear hybrids by introducing an ancestral mtDNA or nuclear genome into the evolved strains. From a combination of growth, competition, and enzymatic assays, along other cellular properties, we should identify which of the nuclear or mitochondrial genome is responsible for compensation. As an example, crossing evolved ND individuals with their ancestor, we could determine if evolved N nuclear genomes converged toward D or if rather the D mitochondrial genome converged toward N. This experiment would provide an additional test for the standing model of mitonuclear evolution, potentially providing experimental support.

Our sequencing efforts have identified several mutant loci, with potential relevance to mitochondrial biology and mitonuclear evolution (see supplementary text S1, Supplementary Material online). Detailed scrutiny of the cell and molecular biology of adaptive mutations at these loci should provide insight into their underlying mechanisms, shedding light not only on paths of mitonuclear compensation, but also fundamental cellular processes. For example, aneuploidies at chromosome IV have been associated with increased gene dosage at *TSA2* in response to oxidative stress (Linder et al. 2017). We could therefore study the impact of overexpression of this gene in various mitonuclear backgrounds, testing for differential effects. Similarly, artificial expansion of the rDNA locus, as in Kwan et al. (2016), may help investigate the adaptive influence of chromosome XII copy number on mitonuclear homeostasis. Long read sequencing at *HPF1* and *MNN4* loci in individuals mutant would provide an accurate map of the associated CNVs. This would enable their reconstitution in various backgrounds. Along with already available *RIM20* knockouts, the expression of altered *HPF1* and *MNN4* constructs could help to study cell wall mannoproteins. We could further study their role in yeast buoyancy, in addition to their impact on respiration, oxidative stress, and chronological lifespan.

### Summary and Conclusion

In this study, we used experimental evolution of yeast mitonuclear hybrids and whole-genome sequencing of the resulting individuals to uncover patterns of evolutionary convergence specific to unique mitonuclear backgrounds. We also confirmed that phenotypic outcomes of evolution are primarily contingent on the nuclear background, with a modulating influence for mitonuclear interactions. We further showed that mitonuclear interactions exert an influence on the molecular mechanisms of adaptation, potentially constraining and potentiating long-term evolution. To our knowledge, this study represents the first direct experimental demonstration of mitonuclear interactions inflecting the evolutionary trajectories of adapting populations. In addition to the support provided to standing evolutionary theory, the strains generated in this study and the accumulated data provide material for further studies of mitonuclear evolution and the cell biology of yeast.

## Materials and Methods

Detailed methods are provided in supplementary text S1, Supplementary Material online.

### Assessing the Growth of Evolved Individuals on Fermentable and Nonfermentable Solid Media

Highly replicated assessment of growth was performed for ancestral strains and evolved individuals on fermentable and nonfermentable solid medium. Strains were printed as 1,536 arrays at randomized positions on YPD or YPG agar omnitrays using robotically manipulated pin tools (BM5-SC1, S&P Robotics, Toronto, ON, Canada). Growth at 30 °C was monitored by recording photographs of the plates at 2-h intervals in a spImager custom robotic platform (S&P Robotics). Colony sizes from pictures were measured with R package Gitter (Wagih and Parts 2014). Growth curves were drawn for each colony and fitted to a modified Gompertz model (Zwietering et al. 1990) to extract estimates of the maximum specific growth rate (*µ*) and carrying capacity (*A*).

### Enzyme Assays on Whole Cell Extracts from *S. cerevisiae*

Initial precultures in 2XYPD were inoculated from glycerol stocks. After a 24-h incubation at 30 °C, precultures were used to inoculate YPG cultures, which were propagated for 24 additional hours. This second preculture was used to inoculate a larger YPG culture, which was propagated until it reached *A*_600_ = 0.5–1.0. Cells were pelleted by centrifugation and frozen overnight at −80 °C. Thawed pellets were suspended in an ice-cold lysis buffer and bead-beaten repeatedly. Whole-cell lysates were frozen at −80 °C until use. Enzyme activities were determined spectrophotometrically using a Mithras LB940 microplate reader (Berthold technologies, Germany) and data analyzed with MikroWin 2010 software (Labsis Laborsysteme, Germany). Enzymatic capacities were expressed as mU·mg protein/s (U·mg protein/s in the case of CAT), where *U* refers to 1 *µ*mol of substrate transformed to product per minute. Chemicals were purchased from Sigma-Aldrich (Oakville, ON, Canada). Enzymatic assays were performed at 30 °C. Detailed assay conditions for each enzyme are provided in supplementary text S1, Supplementary Material online.

### Experimental Evolution

Experimental evolution and related experiments were performed in standardized microtiter culture vessels, termed evolution plates, as described in supplementary text S1, Supplementary Material online. Individual colonies from each of 7 mitonuclear backgrounds were used to inoculate 96 cultures. These cultures were propagated in either YPD (fermentable) or YPG (nonfermentable) media by robot-assisted daily serial passage for 58 days, saving glycerol stocks at 7-day intervals. Hence, 1,344 evolving populations were launched at the onset of the experiment. A single, independently evolved individual was isolated from end point cultures of each evolving population by streaking for single colonies.

### Whole-Genome Sequencing of Evolved Individuals and Ancestral Strains

Genomic DNA was extracted from yeast using a custom protocol based on the DNeasy Blood and Tissue Kit (Qiagen, Venlo, Netherlands) in a 96-well format. Extracted nucleic acids were treated with RNase A and further purified with SPRI beads. DNA concentration was assessed fluorometrically. Sequencing libraries were prepared by tagmentation with the addition of custom DNA barcodes. Equimolar amounts of all libraries were pooled and sequenced on an Illumina NovaSeq6000 S4 PE150 sequencing lane.

### Analysis of Sequencing Data

Quality control and read mapping pipeline was as follows: quality control with FastQC (Andrews 2010), adapter trimming with Trimmomatic (Bolger et al. 2014), merger of overlapping read pairs with BBMerge (Bushnell et al. 2017), primary read mapping with bwa mem (Li and Durbin 2009), and indel realignment with GATK3 (McKenna et al. 2010; van der Auwera and O’Connor 2020). Metrics were collected with picard and samtools (Li et al. 2009; Broad Institute 2016). Whole genome pileups were extracted with samtools. The fraction of coding sequence bases mapped to the mitochondrial genome was computed from pileup, summing the depth of coverage with base quality greater than 20. This sum across the mitochondrial genome divided by the same sum over the whole genome provided an estimate of the fraction of mitochondrial DNA in each cell. Aneuploidies were called from pileup, identifying chromosomes with mean depth of coverage significantly higher or lower than the rest of the genome. Copy number variants (CNVs) were called according to Yoon et al. (2009). Potential artefactual CNVs were resolved with the help of multiple linear models based on depth at locus specific positions. Primary SNV calling was performed using GATK HaplotypeCaller and a custom pipeline computed from pileup. Multiple additional filters were applied to identify the final list of SNVs. CNV and SNV calls were annotated using YeastMine (Balakrishnan et al. 2012).

### Analysis of Dissimilarity between Mutational Profiles

Mutational profiles were built for each combination of mitonuclear background and evolution regimen (evolutionary circumstances) by counting mutations associated with each mutant annotation. Bray–Curtis dissimilarity was computed between the mutational profiles of all pairs of evolutionary circumstances. To avoid ascribing arbitrary or disproportionate evolutionary importance to certain mutations based their size in base pairs, dissimilarity was calculated giving equal weights to all mutation types (aneuploidies, CNVs, and SNVs). Nonparametric multidimensional scaling was performed from the resulting dissimilarity matrix, preserving the best from 10,000 independent initializations of the algorithm. The algorithm was run for a maximum of 3,000 iterations, declaring convergence using a stress tolerance threshold of 10^−9^.

### Specificity of Mutant Loci for Carbon Sources and Nuclear and Mitochondrial Backgrounds

The same strategy was applied to test the specificity of mutant loci for carbon source, nuclear background, mitochondrial background, and all combinations thereof. For each locus, a contingency table was assembled reporting counts of mutations within and outside the locus in each category. This table was submitted to a *χ*^2^ test of independence, yielding a *P* value. Mutual information (MI, reported as dits) was calculated from the same contingency table. Significance thresholds on *χ*^2^*P* values were corrected for false discovery rate on all loci. Loci with *χ*^2^*P* value below threshold and mutual information two standard deviations above mean MI were deemed nonrandomly distributed between categories and thus displaying specificity.

### Competition Assays between Knockouts and Their Ancestral Strains

Changes in fitness incurred by deletion at loci of interest were quantified by performing competition assays in fermentable and nonfermentable media, using a method inspired by Breslow et al. (2008). Ancestral and mutant derivatives marked with different fluorescent markers were mixed and cultured for 3 days, measuring their relative abundance daily by flow cytometry. The effect of loss-of-function alleles on fitness was inferred by linear regression on time series of relative abundance of ancestral and mutant cells.

## Supplementary Material

msad061_Supplementary_Data
