# Supplementary material for: Evolutionary Trajectories are Contingent on Mitonuclear Interactions
Source: Mol Biol Evol. 2023 Mar 17;40(4):msad061. doi: 10.1093/molbev/msad061 (PMC10072823; doi:10.1093/molbev/msad061)
Supplement: msad061_Supplementary_Data [file msad061_supplementary_data.zip › Text S1.pdf]

Supplementary text

Supplementary discussion

Limitations of the study

Our evolution experiment lasted approximately 300 generations. While published experimental evolution studies of a few hundred generations are common, this remains short on the evolutionary time scale. For comparison, this would correspond to roughly 7500 years of human evolution. While most studies cannot realistically last for tens of thousands of generations, a few thousand generations are commonplace (Lenski 2017). The typical workaround for short-term experimental evolution is increased replication (Mcdonald 2019). With more than 1300 starting populations, our experiment certainly meets the high replication standard, yet studies with similar levels of replication have reported lengths of up to a thousand generations (Lang et al. 2011; Lang et al. 2013). Despite this limitation, we were able to identify numerous and clear patterns of evolutionary convergence within genotypes. In addition, we could assign both mitonuclear and carbon source specificity to many mutant loci. Furthermore, it is uncertain that prolonged evolution would have increased the statistical power of our study. While it may have exacerbated patterns of specificity at some loci, over long periods, the accumulation of fixation events coupled to clonal interference and negative epistasis could have reduced the diversity of detectable patterns. However, it could also have enabled the detection of patterns that could only emerge after the fixation of earlier mutations. In short, by affording time for divergence, a longer evolution may have helped observe more sharply defined evolutionary trajectories for each mitonuclear background.

By evolving yeast in two environments that differed solely by the identity of the available carbon source, we hoped to test for the effect of explicitly selecting for a mitochondrial function, while minimizing confounding factors. In retrospect, comparing outcomes of evolution in two highly similar environments may have led to excessively similar outcomes, especially at the phenotypic level. Variation of an additional factor, such as nitrogen source, temperature, or even carbon source concentration, may have helped further differentiate populations evolving in distinct conditions, and better identify effects specific to mitochondrial function. Nevertheless, our experimental setup proved sufficient to identify carbon source-specific mutational patterns and compare patterns of divergence associated with different selective pressures.

The seven mitonuclear backgrounds that we submitted to experimental evolution were derived from just three original strains. We screened our collection of cytoductants for mitonuclear mismatches with readily detectable phenotypic differences, maximizing our chances of observing bona fide examples of mitonuclear

interactions. This may have unduly restricted the breadth of observable patterns of mitonuclear interaction and evolution, biasing our results towards specific evolutionary outcomes. An alternative would have been to propagate a much wider diversity of mitonuclear backgrounds, potentially evolving our entire collection. This agnostic approach would have limited potential artefactual biases induced by the experimental design, at the expense of per background replication.

Most of the evidence presented in this study relies directly or indirectly on the results of sequencing. We did phenotype each of our evolved isolates. We also attempted recapitulation of some of the loss-of-function alleles identified by sequencing, generating corresponding knockouts in ancestral backgrounds. Yet, these experiments either imperfectly mimicked the conditions of evolution, were limited in scope, or relied on simplifying assumptions. Indeed, systematic assessment of growth kinetics in evolved isolates was performed on solid medium for ease of replication and automation, whereas evolution was performed in liquid to accelerate evolution. Thus, phenotypic insight from growth on solid may not accurately reflect growth in the conditions of evolution. In contrast, high-sensitivity competition assays on recapitulated loss-of-function alleles assessed fitness effects in the exact conditions used for evolution. Yet, those assays concerned but a limited number of loci. For technical reasons, mutations to the mitochondrial genomes and aneuploidies had to be excluded from these tests. More generally, our restricted choice of loci may not reflect most mutations, biasing our view of fitness effects. Potentially more impactful is our simplifying assumption that mutations at the chosen loci were pure loss-of-function alleles, which we chose to recapitulate using whole gene knockouts. Careful reconstitution of the exact mutations identified by sequencing, while less scalable, may have provided a more accurate view of fitness effects. Finally, knockouts were prepared in ancestral backgrounds, ignoring epistatic effects incurred by other mutations accumulated in evolved individuals. We thus acknowledge that our phenotypic data represents an approximation, potentially complemented by more targeted future studies.

Significance of identified genome changes

By sequencing the genomes of evolved individuals, we identified changes in almost 2000 genomic features, from the whole chromosome to single gene scales. Features that were the target of a strong evolutionary convergence signals are obvious candidates for functional scrutiny, as were patterns of mitonuclear and environmental specificity. Statistically significant association with changes in phenotype represents another trait of interest. In this section, we discuss some of the most prominent annotations according to these considerations, as they may relate to mitochondrial biology and mitonuclear interactions.

<u>*AMN1* and aneuploidies at chromosomes IV, IX and XII</u>

In yeast, aneuploidy and other chromosome-scale rearrangements have been involved in response to several environmental stresses (Gilchrist and Stelkens 2019). On the macroevolutionary scale, aneuploidies are believed to allow rapid changes in gene dosage upon exposure to stress. They tend to be transient, affording time for finer, more targeted and less costly mutations to be selected, and this is the role these aneuploidies most likely played in our evolution experiment. Aneuploidies at chromosomes XII and IV are both compatible with a general response to poorly functioning mitochondria. Increased copy number for chromosome IV, exclusively observed in backgrounds NN and ND in our experiment, is the least frequent aneuploidy in natural *S. cerevisiae* populations. Because it is the largest in budding yeast, additional copies of chromosome IV represent a hefty fitness cost (Torres et al. 2007; Peter et al. 2018), which may explain the relative scarcity of this evolutionary outcome compared to aneuploidy for chromosomes IX and XII. Copy number changes at chromosome IV has nevertheless been associated with adaptation to oxidative stress, probably increasing the number of copies of gene *TSA2*, a thioredoxin peroxidase involved in the removal of reactive oxygen species (Linder et al. 2017), the production of which would be expected to increase in cells with a poorly tuned electron transport chain. Chromosome XII is notable for carrying the highly repetitive rDNA locus. Changes in the size of the rDNA locus has been involved in response to DNA replication stress (Salim et al. 2017), again as would be expected from cells submitted to ROS-induced DNA damage. At the other end of the spectrum, aneuploidy at chromosome IX is among the most frequent in yeast (Peter et al. 2018) and has been involved in resistance to hygromycin B. In our experiment, it occurs in almost all mitonuclear backgrounds, but preferentially in hybrids DN and DY, which share the same nuclear background but bear mismatched nuclear and mitochondrial genomes. Perhaps related to these large-scale genomics changes, gene *AMN1* encodes for a protein implicated in the regulation of cell-cycle progression (Fang et al. 2018), specifically involved in daughter cell separation and mitotic exit. Overexpression of *AMN1* is known to result in chromosomal instability (Ouspenski et al. 1999), and thus loss-of-function at this locus may promote adaptive aneuploidies and other chromosomal rearrangements in highly stressed populations.

<u>*HPF1*, *MNN4*, *RIM20* and *RIM101*</u>

We repeatedly identified CNVs in gene *HPF1* affecting the N-terminal and C-terminal ends of the protein, the vast majority found in isolates of background ND. A relationship between *HPF1* and respiratory function has been described. Expansion of intragenic repeats in this gene is associated with cell buoyancy, which improves access to oxygen and thus promotes growth in non-fermentable conditions. Increased exposure to oxygen in *HPF1* mutants is also associated with a shortened lifespan, probably caused with heightened oxidative stress (Barré et al. 2020). Although this information would predict preferential selection for *HPF1*

alleles in non-fermentable conditions, we almost exclusively found mutations mapping to this gene in individuals evolved in fermentable medium. While we do not explain this discrepancy, hints from the literature nevertheless indicate a clear connection to mitochondrial function. *HPF1* encodes for a cell wall mannoprotein, and another locus of note in our dataset associated with a strong signal of evolutionary convergence is *MNN4*, a putative regulator of cell wall protein mannosylation (Odani et al. 1997). As for *HPF1*, almost all *MNN4* mutations are CNVs, affecting the C-terminal end of the encoded protein. Chiefly selected in non-fermentable medium, the majority of *MNN4* mutations were identified in individuals bearing the Y nuclear background. Interestingly, *MNN4* has a paralog, *MNN14*, that is repressed by *RIM101* (Conde et al. 2003), another minor convergent hit identified among our evolved individuals. *RIM101* is in turn proteolytically activated by *RIM20*, a prominent, fermentable medium specific site of evolutionary convergence in our experiment. In our data, *RIM20* is rife with nonsense mutations, suggesting a loss-of-function mechanism. We recapitulated this loss of function by performing knockouts at *RIM20* in parental strains, yielding strong gains in fitness in both fermentable and non-fermentable media, especially in backgrounds most affected at this locus. We thus propose that mutations at loci *MNN4*, *RIM20* and *RIM101* are associated with increased cell wall protein mannosylation, in a manner potentially related to *HPF1* function, cell buoyancy and aeration.

COX1 and *COX3*

COX1 and *COX3* are mitochondrial genes, and both encode for subunits of cytochrome *c* oxidase, an integral membrane protein complex catalyzing the final step in the electron transport chain (Cooper et al. 1991). Mutations to both genes are most often observed in background DY evolved in non-fermentable medium. One mutation in exon 11 of *COX1*, causing an alanine to asparagine substitution, occurs recurrently in our evolved isolates. The role of these genes in a critical mitochondrial process within a complex of both nucleus- and mitochondrion-encoded genes identify them as the most likely candidates in our dataset for mitonuclear compensatory mutations.

SIR2, *SIR3* and *SIR4*

SIR3 and *SIR4*, via interactions with sirtuin family gene *SIR2*, are involved in chromatin silencing (Swygert et al. 2018). All three genes have been implicated in yeast cell aging and silencing of rDNA (Kennedy et al. 1997; Sinclair et al. 1997; Park et al. 1999). Yeast *SIR2* is also a mediator of mitophagy (Sampaio-Marques et al. 2012). In mammalian cells, sirtuins have been directly implicated in mitonuclear communication in an NAD-mediated manner (Janssen et al. 2019). While *SIR4* mutations are significantly associated with nuclear background D, they are encountered in both carbon sources and most mitonuclear backgrounds. *SIR2* and *SIR3* mutations display specificity for neither carbon source nor background. A sirtuin-mediated mechanism

of adaptation may thus be a general feature of our evolution experiment and may not bear any direct relevance to mitonuclear biology and evolution. For example, expansion of rDNA arrays at chromosome XII are known inhibitors of *SIR2* (Michel et al. 2005). Aneuploidies at chromosome XII and hitherto undetected expansions of rDNA in evolved individuals may therefore have exerted a selective pressure on *SIR* genes.

<u>*NUM1*</u>

NUM1 mutations are recurrent in our dataset, but do not display specificity. The role of *NUM1* in mitochondrial biology is well described. As the core component of the mitochondria–ER cortex anchor, *NUM1* is essential to both the distribution and function of mitochondria (Ping et al. 2016). Its role is to tether mitochondria to the plasma membrane. Disruption of this function leads to defects in mitochondrial fission and fusion, unbalanced distribution of mitochondria between mother and daughter cells, and reduced lifespan (Cerveny et al. 2007; McFaline-Figueroa et al. 2011; Pernice et al. 2018).

Detailed methods

Culture media. Below are the recipes for the media used in this study. Medium ingredients were purchased from BioShop (Burlington, ON, Canada). Experimental evolution, with its starter cultures, as well as growth and competition assays were all performed in media assembled from ingredients of the same production lot (see below).

Yeast peptone dextrose (YPD). 10 g/L yeast extract, 20 g/L tryptone, 20 g/L glucose. 2XYPD is assembled by doubling the concentration of all components in YPD.

Yeast peptone glycerol (YPG). 10 g/L yeast extract, 20 g/L tryptone, 20 g/L glycerol.

Yeast peptone ethanol glycerol (YPEG). 10 g/L yeast extract, 20 g/L tryptone, 30 g/L ethanol, 20 g/L glycerol.

Enhanced YPD (eYPD). 15 g/L yeast extract, 40 g/L tryptone, 40 g/L glucose, 5 g/L malt extract, 2.5 g/L glycerol, 0.4 g/L L-cysteine hydrochloride, 0.83 g/L K_2HPO_4, 3 g/L Tris pH 6.0.

Amino acid dropout without uracil and without histidine. The following compounds were mixed in powder form: 0.5 g L-adenine sulfate dihydrate, 2 g L-arginine hydrochloride, 2 g L-aspartic acid, 2 g L-glutamate monosodium salt, 10 g L-leucine, 2 g L-lysine monohydrochloride, 2 g L-methionine, 2 g L-phenylalanine, 2 g L-serine, 2 g L-threonine, 2 g L-tryptophan, 2 g L-tyrosine, 2 g L-valine.

Enhanced synthetic complete (eSC). 3.48 g/L yeast nitrogen base without ammonium sulfate and without amino acids, 2 g/L monosodium glutamate, 40 g/L glucose, 2.5 g/L glycerol, 0.4 g/L L-cysteine

hydrochloride, 2.4 g/L amino acid dropout without uracil and without histidine, 0.83 g/L K_2HPO_4, 3 g/L Tris pH 6.0.
Solid medium was made by adding 1.5%-2% agar to the above recipes.

Screen of collection of yeast cybrids. A collection of yeast cybrids was previously constituted, as described in (Paliwal et al., 2014; Wolters et al., 2018). It contains all possible combinations of nuclear and mitochondrial genomes from a set of fifteen strains of *S. cerevisiae*, in four biological replicates. Each strain in the collection was assayed for growth in non-fermentable YPG medium as follows. All manipulations were performed following standard sterile technique. Five microliters from thawed glycerol stocks of the collection were transferred to YPD evolution plates and incubated for 48 hours at 30°C, 65% relative humidity (RH), with shaking at 165 rpm in a microplate incubator shaker (Multitron, Infors HT, Basel, Switzerland). With the help of a liquid handling robot (Freedom Evo, Tecan, Männedorf, Switzerland), 5 μL from these prolonged cultures were transferred to the wells of a flat bottom polystyrene microtiter plate (Greiner, Kremsmünster, Austria), each well containing 235 μL of YPG and a 2.5 mm diameter glass bead. This second plate was covered with a transparent lid, with sides wrapped in three layers of parafilm (Bemis, Sheboygan Falls, WI, USA). Plate was placed in a Tecan Infinite M Nano microplate reader and incubated for 48 hours at 30°C, recording A_{595} at 20 min intervals. Each 20 min interval consisted of 13 min orbital shaking (6 mm diameter), 5 min absorbance measurement, and 2 min resting time. Raw growth curve data was fitted to a modified Gompertz model, as in (Zwietering et al., 1990). Area under the fitted curve was calculated using the trapezoidal rule.

Evolution plates. Experimental evolution and related experiments were performed in standardized microtiter culture vessels, assembled as follows. Flat-bottom polypropylene microtiter plates, in 96 well format (cat. nb. 951040005, Eppendorf, Hamburg, Germany) served as reusable, autoclavable culture vessels. A clean 2.5 mm diameter glass bead (Biospec, Bartlesville, OK, USA) was placed in each well. Wells were filled with 240 μL media, then sealed with an adhesive foil seal (VWR, Wayne PA, USA). Plates were next covered with clean polypropylene lids held with autoclave tape. Plates were sterilized with a 20 min liquid autoclave cycle at 121°C. Plates were left at room temperature to cool and dry. Plates were then wiped of any residual water, placed in airtight plastic bags, and stored at 4°C until use. Before use, plates were equilibrated at room temperature and centrifuged at 2000 rpm for 2 min. Immediately before inoculation, lids were removed, and foils seals discarded. Following inoculation, plates were sealed with breathable adhesive membranes (VWR, Wayne PA, USA), and covered with polypropylene lids held with masking tape at all four corners. For re-use, plates were emptied, collecting and rinsing beads. Beads were sanitized by a 1 hr+ incubation in warm 10% bleach, rinsed thoroughly with tap water, and dried at 56°C in

a Pasteur oven. Plates were washed in soapy water, rinsed thoroughly with tap water, and left inverted at room temperature until fully dry.

Assessing the growth of ancestral and evolved individuals on fermentable and non-fermentable solid media. High-throughput assessment of growth in evolved individuals and their ancestral strains was performed by recording the growth of colonies on YPD and YPG agar medium. Replication and array of samples in preparation for growth curves were performed with robotically manipulated pin tools (BM5-SC1, S&P Robotics, Toronto ON, Canada). Thawed glycerol stocks were replicated from 96-well plates to omnitrays (Nunc, Rochester, NY, USA) in 96-array format on YPD agar medium. Replicas were incubated at 30°C until large, well-defined colonies were visible. Replicas were then cherry-picked and re-arrayed to 384-array format on YPD agar, randomizing positions. Following outgrowth, plates were combined into 1,536-array format on YPD agar omnitrays. Evolved individuals of a given mitonuclear background, evolved in both fermentable and non-fermentable conditions, were printed on a single 1,536 array. Each evolved individual was printed as six replicates. Hence, one 1,536 array was prepared per mitonuclear background for a total of seven arrays, whereas nine replicates of each of the ancestral strains were printed onto all seven arrays. To avoid border effects, top and bottom two rows of the arrays were printed with a filler strain, as were the two extreme left and right columns, and these were excluded from analysis. Next, these randomized arrays in 1,536 format were outgrown, then replicated on YPD and YPG agar. The replicas were incubated at 30°C for 48 hrs (YPD) or 72 hrs (YPG) in a spImager custom robotic platform (S&P Robotics), recording photographs of the plates at 2-hour intervals. The outgrown replicas were replicated to fresh plates for two additional rounds of growth. Data from the second and third rounds was retained for analysis.

Analysis and quantitation of growth on solid medium. Colony sizes from pictures were measured with R package Gitter (Wagih and Parts, 2014). Growth curves were drawn for each colony, and fitted to a modified Gompertz model, as described above, extracting estimates of the maximum specific growth rate (μ) and carrying capacity (A). Ancestral controls were used to estimate plate-specific effects on growth parameters and to normalize estimates across arrays. Specifically, the effect of a given plate on a growth parameter was calculated by dividing each individual ancestral estimate by the geometric mean of the ancestral estimates over all plates in each carbon source, then extracting the median of that ratio over all ancestral estimates from the same plate. The normalization factor thus obtained was used to multiply all estimates from a plate. True value for growth parameters of any given strain was estimated using the median (if $n \geq 25$) or geometric mean (if $n < 25$) over all replicates, with confidence intervals obtained by bootstrapping. A series of two-way ANOVAs with interaction was performed on growth data with nuclear

and mitochondrial backgrounds as factors, Separate ANOVAs were conducted for each combination of fitness proxy, evolution regimen, and carbon source.

Preparation of whole extracts from strains of *S. cerevisiae* for enzyme assays. Precultures were inoculated by transferring 5 μL from thawed glycerol stocks to 5 mL 2XYPD medium. Cultures were incubated with shaking at 30°C for 24 hrs. Half a milliliter from precultures was used to inoculate 5 mL YPG, and this new culture was incubated with shaking at 30°C for 24 hrs. This second culture was diluted in 45 mL of pre-warmed YPG medium. This subculture was incubated at 30°C with shaking until it reached A_{600}=0.5-1.0. Cells were pelleted by centrifugation, then suspended in water. Cells were pelleted once again by centrifugation, washed in 25 mM potassium phosphate pH 8.0, then frozen as pellets at -80°C. After overnight freezing, pellets were thawed on ice, and suspended in 0.5 mL of freshly prepared, ice-cold 25 mM Tris-HCl pH 7.5, 1 mM EDTA, 100 mM NaCl + cOmplete Mini, EDTA-free Protease Inhibitor Cocktail (Roche, Basel, Switzerland). Suspension was bead beaten at 4°C for 10 cycles of 2 min beating, 2 min rest on ice. Lysate was frozen at -80°C.

Enzyme assays on whole cell extracts from *S. cerevisiae*. Enzyme activities were determined spectrophotometrically using a Mithras LB940 microplate reader (Berthold technologies, Germany) and data analyzed with MikroWin 2010 software (Labsis Laborsysteme, Germany). Enzymatic capacities were expressed as mU·mg $proteins^{-1}$ (U·mg $proteins^{-1}$ in the case of CAT), where U refers to 1 μmol of substrate transformed to product per minute. Chemicals were purchased from Sigma-Aldrich (Oakville, Ontario, Canada). Enzymatic assays were performed at 30°C in the following conditions:

Malate dehydrogenase (MDH, EC 1.1.1.37). MDH activity was measured at 340 nm for 4 min, following the oxidation of NADH (ε_{340} = 6.22 mM^{-1}·cm–^{1}). The medium consisted of 100 mM potassium phosphate, 0.2 mM NADH and 0.5 mM oxaloacetate (OAA), pH 7.5. The background activity in absence of sample was subtracted from the main results (Bergmeyer, 1983).

Mitochondrial complex I + III (ETS, EC 7.1.1.2 and 7.1.1.8). ETS activity was measured at 490 nm using NADH as electron donor, following the reduction of p-iodonitrotetrazolium violet (INT, ε_{490} = 15.9 mM^{-1}·cm^{-1}) for 6 min. The medium consisted of 50 mM potassium phosphate, 0.85 mM NADH, 2 mM INT and 0.03% (v/v) triton X-100, pH 7.5. The specificity of the reaction was verified in absence of NADH and the residual activity was subtracted from the main results (Hunter-Manseau et al., 2019).

Cytochrome c oxidase (CCO, EC 7.1.1.9). CCO activity was measured at 550 nm following the oxidation of cytochrome c (cyt c, ε_{550} = 18.5 $mM^{-1}\cdot cm^{-1}$) for 6 min. Cyt *c* was reduced through the addition of 4.5 mM dithionite. The medium consisted of 50 mM potassium phosphate, 50 μM cyt c, 1 mM ADP and 0.03% (v/v) triton X-100, pH 7.0. The specificity of the reaction was verified in presence of 40 mM sodium azide (CCO-inhibitor), and the residual activity was subtracted from the main results (Lemaire and Dujardin, 2008; Spinazzi et al., 2012; Hunter-Manseau et al., 2019).

ATP-synthase (ATPase, EC 7.1.2.2). ATPase activity (ATP hydrolysis) was measured at 340 nm following the oxidation of NADH (ε_{340} = 6.22 $mM^{-1}\cdot cm^{-1}$) for 4 min. The medium consisted of 250 mM sucrose, 20 mM HEPES, 5 mM $MgSO_4$, 0.35 mM NADH, 2.5 mM phosphoenolpyruvate (PEP), 2.5 mM ATP, 5 µM antimycin A, 4 units · mL^{-1} lactate dehydrogenase (LDH) and 4 units · mL^{-1} pyruvate kinase (PK), pH 8.0. The specificity of the reaction was verified in presence of 25 µM oligomycin (ATPase-inhibitor), and the residual activity was subtracted from the main results (Barrientos et al., 2009; Haraux and Lombes, 2019; Rodriguez et al., 2020).

Catalase (CAT, EC 1.11.1.6). CAT activity was measured at 240 nm following the disappearance of H_2O_2 (ε_{240} = 43.6 $M^{-1}\cdot cm^{-1}$) for 1 min. The medium was composed of 100 mM potassium phosphate, 0.1% (v/v) triton X-100 and 60 mM H_2O_2, pH 7.5. The background activity in absence of sample was subtracted from the main results (Page et al., 2009; Pichaud et al., 2010; Orr and Sohal, 1992; Bettinazzi et al., 2021; Christen et al., 2020).

Citrate synthase (CS, EC 2.3.3.1). CS activity was measured at 412 nm for 6 min, following the increase in absorbance due to the reaction between 5,5'-dithiobis-(2-nitrobenzoic acid) (DTNB) and CoA-SH to form TNB (ε_{412} = 14.15 $mM^{-1}\cdot cm^{-1}$). The medium was composed of 100 mM Tris-HCl, 0.1 mM DTNB, 0.1 mM acetyl-CoA (AcCoA), 0.15 mM oxaloacetate (OAA), pH 8.0. The specificity of the reaction was verified in absence of OAA and the residual activity subtracted from the main results (Spinazzi et al. 2012; Hunter-Manseau et al. 2019).

Mitochondrial complex II (SDH, EC 1.3.5.1). SDH activity was measured at 600 nm using succinate as electron donor and following the reduction of 2,6-dichloroindophenol (DCIP, extinction coefficient ε_{600} = 19.1 $mM^{-1}\cdot cm^{-1}$) for 6 min. To fully activate the enzymatic complex, samples were incubated for 10 min at the assay temperature (30°C) in a reaction medium consisting of 50 mM potassium phosphate, 20 mM succinate and 5 mM $MgCl_2$, pH 7.5. The reaction was started by addition of 50 μM DCIP, 65 μM ubiquinone1 (CoQ1), 4 μM antimycin A, 2 μM rotenone and 10 mM sodium azide. The specificity of the

reaction was verified in absence of CoQ1 and the residual activity subtracted from the main results (Barrientos et al. 2009; Breton et al. 2009; Spinazzi et al. 2012; Hunter-Manseau et al. 2019).

Protein content. Protein concentration ($mg \cdot mL^{-1}$) was determined with the BCA assay (Smith et al., 1985).

Preparation of starter cultures for experimental evolution. All procedures were performed under laminar flow within a sterile enclosure. This protocol was performed identically for strains of each of the following mitonuclear backgrounds: $273614N^{273614N}$ (NN), $273614N^{DBVPG6044}$ (ND), $DBVPG6044^{273614N}$ (DN), $DBVPG6044^{DBVPG6044}$ (DD), $DBVPG6044^{Y12}$ (DY), $Y12^{DBVPG6044}$ (YD), $Y12^{Y12}$ (YY). Streaks from each of the four biological replicates from each of the mitonuclear backgrounds were prepared on YPEG agar, and incubated at 30°C for 48 hrs. From each streak, 24 colonies were picked at random with sterile micropipette tips and inoculated into YPD evolution plates. Hence, 96 cultures were launched from each of the seven mitonuclear backgrounds submitted to experimental evolution. Plates were incubated overnight at 30°C, 65% RH with shaking at 200 rpm. Glycerol stocks were prepared by mixing 100 μL from the starter cultures with 100 μL 75% glycerol in the wells of conical bottom polypropylene plates (Greiner, Kremsmünster, Austria). Remainder of the cultures were used immediately to launch experimental evolution.

Experimental evolution on mitonuclear hybrids of *S. cerevisiae*. Our collection of yeast cybrids contains four biological replicates of each mitonuclear background, each derived from a separate cybridization event. Twenty-four evolutions were founded from each of these cybrids, hence 96 replicate populations of each of seven mitonuclear backgrounds were propagated by daily serial passage in both fermentable (YPD) and non-fermentable (YPG) medium for 58 days. All procedures were performed under laminar flow within a sterile enclosure. Starter cultures were used to inoculate a first batch of experimental evolution cultures. With the help of a liquid handling robot (Freedom Evo 150, Tecan, Männedorf, Switzerland), 5 μL from each starter culture were transferred to YPD evolution plates, and in parallel to YPG evolution plates. Tips used for transfer were washed with sterile water and kept for further transfer of the same cultures. Plates were incubated for 24 hrs at 30°C, 65% RH with shaking at 200 rpm. Evolving populations were then transferred to new evolution plates for 58 consecutive days. On day 2 and every 7 days afterwards until day 58, glycerol stocks were prepared by mixing 100 μL from the evolving populations with 100 μL 75% glycerol in the wells of conical bottom polypropylene plates.

Isolation of independently evolved individuals. Glycerol stocks from day 58 of the evolution experiment were thawed at room temperature. Ten microliters from each stock were transferred to the surface of a YPD

agar petri dish. The resulting drop from the glycerol stock was streaked for single colonies. Petri dishes were incubated for 48 hrs at 30°C. From each Petri dish, a single colony was picked at random using a sterile tip and placed into a YPG evolution plate. Plates were incubated for 24 hrs at 30°C, 65% RH with shaking at 200 rpm. Glycerol stocks were prepared by mixing 100 μL from the starter cultures with 100 μL 75% glycerol in the wells of conical bottom polypropylene plates (Greiner, Kremsmünster, Austria).

Preparation of total DNA from evolved individuals and ancestral strains. Total DNA from each individual evolved strain and all ancestral strains was extracted and purified as starting material for the preparation of next-generation sequencing libraries. A custom protocol using the DNeasy Blood and Tissue Kit (Qiagen, Venlo, Netherlands) was applied in 96-well format. Each strain was inoculated from glycerol stock in 4 x 1 mL YPD medium, in a polypropylene 96-well deep well plate (DWP). Each DWP was covered with a breathable adhesive membrane and incubated for 24 hours at 30°C with shaking at 200 rpm. Cells were harvested by centrifugation for 10 min at 5000 x g and suspended in 250 μL water. Suspensions of each strain were pooled into a single well, and centrifuged once more for 10 min at 5000 x g. Pellets were suspended in 600 μL of 1 M sorbitol, 100 mM sodium EDTA, 14 mM β-mercaptoethanol, supplemented with 6 U zymolyase (Bioshop, Burlington ON, Canada), then transferred to collection microtubes provided with the DNeasy kit, and incubated at 30°C for at least 30 min. Resulting spheroplasts were pelleted by centrifugation for 10 min at 5000 x g, suspended in 180 µl Buffer ATL + 20 µl proteinase K solution, and mixed thoroughly. Lysis suspensions were incubated at 56°C for at least 30 min, mixing occasionally to disperse the samples. Samples were shaken vigorously for 15 s, then centrifuged briefly to collect all liquid at the bottom of tubes. Samples were supplemented with 4 µL of 100 mg/mL RNase A, mixed vigorously, and incubated at room temperature for at least 5 min. A 50:50 mix of buffer AL and ethanol was prepared fresh, and 400 µL were dispensed to each tube, mixing once again by vortexing. Lysates (max 900 µL) were applied to DNeasy 96 purification columns. Columns were centrifuged for 10 min at 3800 x g. Columns were washed with 500 µL buffer AW1, centrifuging for 5 min at 3800 x g. Columns were further washed with 500 µL buffer AW2, centrifuging for 15 min at 3800 x g. Columns were next placed on a clean rack of elution microtubes and 100 µL of buffer AE were added to each sample. Samples were incubated for at least 1 min at room temperature, then centrifuged for 2 min at 3800 x g. Each eluate was next supplemented with 4 µL of 10 mg/mL RNase A, agitated vigorously, and incubated at room temperature for 15 min. The RNase-treated eluates were further purified by mixing 50 µL from each sample with 20 µL beads and incubating at room temperature for 5 min. After the bead mixture was applied to a magnet for 60s, liquid was removed from the beads, which were washed twice with 200 µL of 80% ethanol. Beads were suspended in 40 µL of 10 mM Tris-HCl pH 8.0 and incubated for 5 min at room temperature. Eluate was separated from beads using a magnet, as described above. Sample quality was evaluated by agarose gel

electrophoresis. Purity and concentration of DNA was assessed by measuring absorbance at 260 nm and 280 nm using a NanoDrop spectrophotometer (ThermoFisher Scientific, Waltham MA, USA). Concentration of DNA in the sample was further estimated using the AccuClear Ultra High Sensitivity dsDNA Quantitation Kit (Biotium, Fremont CA, USA) following manufacturer's instructions.

Preparation of next-generation sequencing libraries. Sequencing libraries were prepared using the Tagment DNA Enzyme and Buffer Large Kit (Illumina, San Diego CA, USA), the KAPA HiFi HotStart ReadyMix 2X (Roche, Basel, Switzerland) and custom DNA barcodes. Genomic DNA samples were diluted with water to a target concentration of 2.5 ng/µL or less. Tagmentation reactions were assembled by mixing 1.25 µL tagment DNA buffer, 0.25 µL tagment DNA enzyme and 1 µL gDNA dilution. Reactions were incubated at 55°C for 5 min. Custom DNA barcodes were added by PCR as follows. Tagmented DNA was mixed with 3.76 µL 2X KAPA ReadyMix, and 0.625 µL both primers. Primers had sequences 5'AATGATACGGCGACCACCGAGATCTACAC-(8-nucleotide custom i5 barcode)-TCGTCGGCAGCGTC3' and 5'CAAGCAGAAGACGGCATACGAGAT-(8-nucleotide custom i7 barcode)-GTCTCGTGGGCTCGG3'. A collection of 32 custom i5 barcodes and 48 custom i7 barcodes was used, enabling the preparation of 1,536 unique barcode combinations. PCR was performed by cycling as follows: 72°C for 3 min, 98°C for 2:45 min, then 8 cycles of 98°C for 15s, 62°C for 30s, 72°C for 3 min, followed by a 1 min final extension at 72°C. Library preparation was completed by reconditioning PCR. Barcoding reactions were mixed with 8.5 µL 2X KAPA ReadyMix, 0.5 µL of 10 µM primer P1 (5'AATGATACGGCGACCACCGA3') and 0.5 µL of 10 µM primer P2 (5'CAAGCAGAAGACGGCATACGA3'). Reactions were then cycled as follows: 95°C for 5 min, then 4 cycles of 98°C for 20s, 62°C for 20s, 72°C for 2 min, followed by a 2 min final extension at 72°C. Final PCR products were diluted with 14 µL PCR grade water, then bead purified as described above, except that only 18 µL beads were added to the PCR reactions. Library concentration was estimated using the AccuClear Ultra High Sensitivity dsDNA Quantitation Kit following manufacturer's instructions. A subset of the libraries was inspected with a BioAnalyzer 2100 using a High Sensitivity DNA chip (Agilent Technologies, Santa Clara CA, USA) for insert size and monodispersity. Equimolar amounts of all libraries were pooled and sequenced on an Illumina NovaSeq6000 S4 PE150 sequencing lane at the Génome Québec Expertise and Service Center (Montréal QC, Canada).

Pre-treatment of sequencing results and alignment to reference genomes. Quality control was performed individually on each sequencing library using FastQC (Andrews, 2010), and results were aggregated using MultiQC (Ewels et al., 2016). Sequencing adapters were trimmed with Trimmomatic (Bolger et al., 2014). Overlapping read pairs were merged using BBmerge (Bushnell et al., 2017). Single

read files from the BBmerge output were concatenated into single files. Primary read mapping to reference genomes was performed using bwa mem (Li and Durbin, 2009). Libraries were mapped to the published genomes of their associated ancestral strains. The *S. cerevisiae* 273614N reference genome sequence was obtained from the diArk database (Hammesfahr et al., 2011) as published in (Liti et al., 2009). Reference genome sequences for *S. cerevisiae* strains DBVPG6044 and Y12 were obtained from NCBI BioProject PRJEB7245, BioSamples SAMEA2757762 and SAMEA2757763, respectively, as published in (Yue et al., 2017). Chimeric reference genomes were assembled for cybrid strains by manually replacing the mitochondrial genome contig published for the nuclear parent's genome with that published for the mitochondrial parent. Alignment was performed separately for merged and paired reads. Hence, BAM outputs from bwa mem were merged and then sorted with samtools (Li et al., 2009). Following the addition of read groups and deduplication with picard (Broad Institute, 2016), indel realignment was performed using GATK3 (McKenna et al., 2010; van der Auwera and O'Connor, 2020). Alignment and depth metrics were collected using picard and samtools, respectively. Whole-genome pileups were extracted for each sequencing library with samtools.

Estimating the fraction of mtDNA per cell. The fraction of sequence bases mapped to coding regions of the mitochondrial genome was used as a proxy for copy number, structural or other changes incurred to the organellar genome. This was computed from pileup, summing the depth of coverage with base quality greater than 20 in known coding regions of the reference mitochondrial genomes. This sum across the mitochondrial genome divided by the same sum over the whole genome provided an estimate of the fraction of coding mitochondrial DNA in each cell.

Identification of aneuploidies from sequencing data. Aneuploidies were called from pileup. Telomeric and subtelomeric regions were excluded from this analysis by masking 25 kb at both ends of each chromosome. High quality depth of coverage was computed at each position by counting reads with both base and mapping quality above 30 on the phred scale. Finally, any position found with average depth of coverage in all biological replicates of the ancestral strains above 5 times the median depth over the whole genome was also excluded from analysis. Next, the ratio between the mean depth of the chromosome and that of the rest of the genome was calculated. If this ratio was between 0.75 and 1.25, a p-value for the mean coverage of the chromosome was computed, assuming normal distribution of depth of coverage across the genome, with mean and standard deviation computed over the rest of the genome. If this p-value was smaller than the length of the chromosome divided by the length of the whole genome, an aneuploidy was suspected. Aneuploidy candidates were confirmed visually by inspecting heatmaps of depth of coverage along the length of chromosomes.

Primary identification of copy number variants (CNVs) from sequencing data. Primary CNV calling was performed essentially according to (Yoon et al., 2009). Analysis was performed from filtered pileups, as described above for aneuploidies. Each chromosome was divided in 100 bp windows, extracting mean depth of coverage for each window. Average and standard deviation on window means were computed across chromosomes, enabling computation of a z-score and p-value for each window. Next, whole chromosomes were scanned with rolling intervals of various window sizes, starting with 2 windows in length. If the maximum p-value for windows within an interval fell below a threshold, determined by both window and chromosome length, the interval was marked as a candidate CNV. Increasing interval sizes were used until a threshold determined by both interval and chromosome length was reached. Overlapping CNV candidates were merged. Any candidate with median depth between 0.75x and 1.25x the median chromosome depth was excluded. A p-value was computed for the mean depth of each candidate, based on mean and standard deviation over windows. If this p-value was greater than 10^{-6}, the candidate was excluded. Finally, if the same area had mean depth of coverage between 0.75x and 1.25x the chromosome-wide median depth in the ancestral strains, the candidate CNV was also excluded.

Resolution of artefactual CNV calls caused by misalignment at homologous loci. Reference genome sequence at CNV coordinates were blasted against the whole reference genome sequence, identifying loci with high homology to CNV candidates. Blast hits with an E-value of less than 1.0, length above 150 bp and percent identity with the CNV locus above 80% were retained for further scrutiny. Sequences at the CNV call and homologous hit loci were aligned with MUSCLE (Edgar, 2004), identifying locus specific positions. A system of linear equations was built from the sums of each base calls at each locus specific positions in the alignment. Multiple linear regression was then used to identify the most likely depth of coverage, with 95% confidence interval, at each homologous locus. If the inferred depth was between 0.75x and 1.25x the chromosome median depth, and the confidence interval on depth at the homologous locus did not overlap with the confidence interval for depth over the whole chromosome, a CNV call was asserted. Otherwise, or if locus specific information was too scarce to lift ambiguity caused by homology, CNV call was not retained. Applying this methodology, several CNVs were rejected, others were retained, while a subgroup was reassigned to more likely loci.

SNV calling. Primary SNV calling was performed using two parallel methods, starting with GATK HaplotypeCaller. SNV calls were combined into a single GVCF file using GATK CombineGVCF. Initial filtering was performed with GATK FilterVariants and SelectVariants. Additional filtering was done with vcftools. A list of SNVs was produced in csv format using bcftools.

In parallel, custom SNV calling was attempted directly from pileup. The following regions of the genome were filtered out of pileups in preparation for SNV calling: high coverage regions in the ancestral strains, subtelomeric regions, positions with mean base and mapping quality below 30, positions with depth of coverage below 10, and positions with N or otherwise missing sequence in the reference. A baseline error model was built, identifying the number of each base call expected at each position, considering the reference base and mean base quality at that position, and the average error rates observed in the data. A chi^2 test for goodness of fit was performed, comparing the expected base counts predicted by the error model with the observed base counts. An E-value was obtained by multiplying this p-value with the number of positions in the genome. Positions with an E-value < 1.0 were retained as potential SNVs, while any position with non-reference base call count less than 25% of total depth was rejected.

The intersection between the results of both primary SNV calling pipelines was kept as the overall primary list of SNVs. SNV calls were additionally filtered for strand, base quality and mapping quality biases. A few libraries were observed to display SNVs that overlapped strongly with those from libraries that were found in close proximity on library preparation plates. Those SNVs were flagged as potential cross-contamination artifacts and removed from further scrutiny. Furthermore, SNVs found in two or more libraries that shared a barcode were tagged as potential index hopping artifacts and removed from SNV calls. Moreover, the same substitutions arising repeatedly at the exact same position were deemed unlikely: any SNV encountered in four libraries or more was rejected. SNVs expected to arise from alignment of reads to the wrong reference genome (e.g. DBVPG6044 reads mistakenly aligned to the Y12 reference genome) were also removed. Next, using Fisher's exact test, SNV calls were identified that were more compatible with the homozygous non-mutant genotype than the heterozygous or homozygous mutant scenarios, and thus rejected. Finally, libraries that presented an unusually high number of SNVs (more than 300 SNVs and more than 2 standard deviations above average number of SNVs per library) were rejected. A heterozygous genotype was called for many SNVs, despite uniformly haploid starting populations. A small sample of evolved individuals were thus assessed for ploidy, as described above, showing diploidization in approximately half sampled isolates (**Fig S16**).

Annotation of CNV and SNV calls. Annotations of the S288c reference genome (Engel et al., 2022) were used for annotating SNV and CNV calls in evolved isolates. Sequences from mutation-carrying loci were blasted against the S288c reference genome to identify corresponding coordinates. Annotations corresponding to these locations were identified with Yeastmine (Balakrishnan et al., 2012).

Analysis of association between phenotypic changes and mutant loci. Quantitative phenotypic data, including fitness proxies (carrying capacity and growth rate) in both fermentable and non-fermentable media, as well as the fraction of mtDNA per cell, are available for each evolved mutant. A list of annotated mutations is also associated with each mutant, enabling tests of the statistical association between mutant loci and phenotypic changes. For each mutant locus and each phenotype in each carbon source, the association was tested as follows. A t-test was performed to determine if the fitness of individuals that display mutations at the locus of interest differed significantly from fitness of their ancestral strains, yielding a p-value. In parallel, using the same data, effect size of mutation at the locus was evaluated by calculating Cohen's *d* corrected for sample size, with associated standard error. P-value of obtaining a given effect size at least as extreme by chance was computed empirically by drawing 10 000 random samples of the same size among mutants of the same mitonuclear background and calculating an effect size for each. These calculations were performed separately for each mitonuclear background since ancestral strains displayed widely differing phenotypes. P-values obtained for each mitonuclear background were combined using Stouffer's method, with the square root of sample sizes used as weights. Significance thresholds were corrected for false discovery rate applying the method of Benjamini and Hochberg on all loci as described in (Kuo, 2017). Loci with both p-values below threshold were deemed significantly associated with quantitative changes in phenotype.

Generation of fluorescent knock-out mutants at putative loss-of-function loci. First, GFP- and mCherry-tagged derivatives of all ancestral strains were generated from three biological replicates. Parental strains all carried the *MATa ho::hphMX ura3::KanMX* genotype. They were transformed with both the GFP and mCherry selection cassettes, which shared the same general structure. The expression of both fluorescent proteins was placed under the control of the *S. cerevisiae TEF2* promoter and *ENO1* terminator. Fluorescent protein expression cassettes were fused to the *natNT2* selection cassette. This full-length cassette was flanked on both extremities with 40 bp homology to the *HIS3* locus and transformed into the ancestral strains using the lithium acetate method. Transformants were selected on eYPD agar medium (as described in *Culture media* subsection above) supplemented with 100 μg/mL hygromycin, 100 μg/mL geneticin and 75 μg/mL nourseothricin. Integration of the fluorescent protein expression cassettes at the *HIS3* locus was confirmed by growth in presence of nourseothricin, loss of histidine prototrophy, detection of a fluorescent signal by flow cytometry and successful PCR amplification at the 5' and 3' junctions between the *HIS3* locus and heterologous sequences. Knockouts of each of the following genes were prepared in all fluorescent derivatives of the ancestral strains: *AMN1*, *GPB2*, *IRA2*, *MNN4*, *RIM20*, *SIR3*, *SIR4*, and *SUC2*. Open reading frames at each of these loci were deleted by transformation with the *Kluyveromyces lactis URA3* expression cassettes amplified from plasmid pUG72 (Gueldener et al. 2002)and

flanked in 5' and 3' with 40 bp homology to the promoters and terminators of the target genes. Transformants were selected on eSC agar devoid of uracil. Knockouts were scored for uracil prototrophy; resistance to hygromycin, geneticin and nourseothricin; detection of a fluorescent signal by flow cytometry and successful PCR amplification at the 5' and 3' junctions between the target locus and heterologous deletion cassette.

Competition assays between knockouts and their ancestral strains. Changes in fitness incurred by deletion at loci of interest were quantified by performing competition assays in fermentable and non-fermentable media, using a method inspired by (Breslow et al., 2008). Knockouts at each of the eight loci of interest, in the mitonuclear background under study, as three biological replicates, labeled with both GFP and mCherry were arrayed as glycerol stocks in 96-well format. Matching arrays of the fluorescently labeled ancestors were also prepared. Precultures in 96-well format of both the ancestral and knock-out strains were inoculated by transferring 5 µL from thawed glycerol stock arrays to YPD evolution plates. These preculture plates were incubated for 24 hrs at 30°C, 65% RH with shaking at 200 rpm. Outgrown precultures of the knockouts and matching ancestral strains were mixed by transferring 5 µL from both to YPD and YPG evolution plates. Control competitions of the mCherry and GFP labeled ancestral strains were also prepared, to account for differences in fitness caused by the fluorescent labels. For three consecutive days following mixing of the strains, competition cultures were propagated and transferred as described above for experimental evolution. On each day, outgrown cultures were diluted 1:50 in non-sterile water. Two thousand cells from each dilution were analyzed by flow cytometry, counting mCherry and GFP positive cells. Flow cytometry measurements were performed with the help of a Guava easyCyte HT (Luminex, Austin TX, USA) or Accuri C6 (BD, Franklin Lakes NJ, USA) instrument. All microbiological procedures were performed under laminar flow within a sterile enclosure, except for endpoint measurement by flow cytometry. All pipetting was performed with the help of a liquid handling robot (Freedom Evo, Tecan, Männedorf, Switzerland). Triplicate competitions were performed on both fluorescent derivatives of all biological replicates in both carbon sources.

Analysis of competition data. The $\log_2$ ratio of mCherry count over GFP count was calculated for each time point in each competing culture. The $\log_2$ ratio at time zero was subtracted from time points of a given competition so that samples may share a common y-intercept. Linear regression was performed on all data points derived from the same biological replicate, fitting to the following model:

$$log_2 \frac{n_{mCherry}}{n_{GFP}} = \Delta\mu_{WTvWT}\mathrm{t} + \Delta\mu_{KO}\mathrm{tM} + \beta_0$$

where $n_{mCherry}$ is the number of mCherry-labeled cells, n_{GFP} is the number of GFP-labeled cells, $\Delta\mu_{WTvWT}$ is the difference in growth rate between the mCherry and GFP labeled ancestor, t is time, $\Delta\mu_{KO}$ is the difference in growth rate between the knock-out and its ancestor, M is a variable with value 0 when a time point belongs to a ancestor vs ancestor competition, and 1 when it belongs to a knock-out vs ancestor competition, and β_0 is a constant. The fit thus provides an estimate for the change in growth rate associated with knocking out the gene of interest in the current mitonuclear background. Standard error (SE) on this coefficient estimate is used to test whether the coefficient is null, and to compute a sum-of-squares error (SS) on the estimate. Estimates for three biological replicates and two fluorescent labels thus provided six estimates for each locus in each mitonuclear background. They were combined into a single estimate by calculating an average weighted by sample size. P-values were combined using Stouffer's method, weighted by sample size. Similarly, sum-of-square errors were combined by summing them and adding the product of sample size with the square deviation of each estimate from the overall mean estimate. Mean fitness effect estimates, and the combined sum-of-squares were used to perform ANOVAs to test if fitness effect of a given locus in each carbon source differed between mitochondrial backgrounds, with accompanying Tukey HSD tests on pairwise differences between backgrounds. Similarly, ANOVAs were performed to determine if fitness effects of loci differed on given mitonuclear backgrounds. Two-way ANOVAs were performed to determine significance of nuclear, mitochondrial, and mitonuclear interaction effects on growth rate changes. Spearman rank correlation was calculated between fitness effects estimated by competition assays and frequency of mutations at the loci in each background, as determined from sequencing data.

Mutational bias. We counted every possible single nucleotide change among all SNV calls in our dataset, and assigned them to their corresponding mitonuclear background. Counts of for each mitonuclear background were compared to the dataset-wide counts applying a chi^2-test of independence, with a p-value threshold of 0.05.

Assessment of ploidy in select strains. Ploidy was determined for all ancestral strains and a subset among evolved individuals, following a method inspired by (Charron et al. 2019). Thawed glycerol stocks of the strains were streaked for single colonies on YPD agar. Single colonies were picked in triplicate and inoculated into 1 mL YPD, in wells of a polypropylene DWP. DWPs were incubated for 24 hrs at room temperature. Cells were washed with 2 x 1 mL water, then fixed for 1 hr in 70% ethanol. Ethanol was removed by washing cells twice with 1 mL water. Cells were next incubated overnight at 37°C in 1 mL of freshly prepared 0.25 mg/mL RNase A. Cells were washed twice with 1 mL of 50 mM sodium citrate pH 7.0. A small volume (25 µL) of the washed cell suspension was diluted in 225 µL of 0.667 µM SYTOX

green (Thermo Fisher Scientific) and incubated in the dark at room temperature for 1 hr. Cell density in the stained sample was adjusted to approximately 500 cells/µL, and analyzed by flow cytometry, recording 5000 events per sample. Mean green fluorescence was measured for cells in G1 phase. All manipulations were performed in triplicate.

References

Barré BP, Hallin J, Yue J-X, Persson K, Mikhalev E, Irizar A, Holt S, Thompson D, Molin M, Warringer J, et al. 2020. Intragenic repeat expansion in the cell wall protein gene HPF1 controls yeast chronological aging. *Genome Res* 30:697–710.

Barrientos A, Fontanesi F, Díaz F. 2009. Evaluation of the mitochondrial respiratory chain and oxidative phosphorylation system using polarography and spectrophotometric enzyme assays. In: Current Protocols in Human Genetics. Vol. 63.

Breton S, Beaupré HD, Stewart DT, Piontkivska H, Karmakar M, Bogan AE, Blier PU, Hoeh WR. 2009. Comparative mitochondrial genomics of freshwater mussels (Bivalvia: Unionoida) with doubly uniparental inheritance of mtDNA: gender-specific open reading frames and putative origins of replication. *Genetics* 183:1575–1589.

Cerveny KL, Studer SL, Jensen RE, Sesaki H. 2007. Yeast mitochondrial division and distribution require the cortical Num1 protein. *Dev Cell* 12:363–375.

Charron G, Marsit S, Hénault M, Martin H, Landry CR. 2019. Spontaneous whole-genome duplication restores fertility in interspecific hybrids. *Nat Commun* 10:4126.

Conde R, Pablo G, Cueva R, Larriba G. 2003. Screening for new yeast mutants affected in mannosylphosphorylation of cell wall mannoproteins. *Yeast* 20:1189–1211.

Cooper CE, Nicholls P, Freedman JA. 1991. Cytochrome c oxidase: structure, function, and membrane topology of the polypeptide subunits. *Biochem Cell Biol* 69:586–607.

Fang O, Hu X, Wang L, Jiang N, Yang J, Li B, Luo Z. 2018. Amn1 governs post-mitotic cell separation in *Saccharomyces cerevisiae*. *PLoS Genet* 14:e1007691.

Gilchrist C, Stelkens R. 2019. Aneuploidy in yeast: segregation error or adaptation mechanism? *Yeast* 36:525–539.

Gueldener U, Heinisch J, Koehler GJ, Voss D, Hegemann JH. 2002. A second set of loxP marker cassettes for Cre-mediated multiple gene knockouts in budding yeast. *Nucleic Acids Res* 30:e23.

Hunter-Manseau F, Desrosiers V, le François NR, Dufresne F, Detrich HW, Nozais C, Blier PU. 2019. From Africa to Antarctica: exploring the metabolism of fish heart mitochondria across a wide thermal range. *Front Physiol* 10:1220.

Janssen JJE, Grefte S, Keijer J, de Boer VCJ. 2019. Mito-nuclear communication by mitochondrial metabolites and its regulation by B-vitamins. *Front Physiol* 10.

Kennedy BK, Gotta M, Sinclair DA, Mills K, McNabb DS, Murthy M, Pak SM, Laroche T, Gasser SM, Guarente L. 1997. Redistribution of silencing proteins from telomeres to the nucleolus is associated with extension of life span in *S. cerevisiae*. *Cell* 89:381–391.

Lang GI, Botstein D, Desai MM. 2011. Genetic variation and the fate of beneficial mutations in asexual populations. *Genetics* 188:647–661.

Lang GI, Rice DP, Hickman MJ, Sodergren E, Weinstock GM, Botstein D, Desai MM. 2013. Pervasive genetic hitchhiking and clonal interference in forty evolving yeast populations. *Nature* 500:571–574.

Lenski RE. 2017. Experimental evolution and the dynamics of adaptation and genome evolution in microbial populations. *ISME J* 11:2181–2194.

Linder RA, Greco JP, Seidl F, Matsui T, Ehrenreich IM. 2017. The stress-inducible peroxidase TSA2 underlies a conditionally beneficial chromosomal duplication in *Saccharomyces cerevisiae*. *G3 Genes|Genomes|Genetics* 7:3177–3184.

Mcdonald MJ. 2019. Microbial experimental evolution - a proving ground for evolutionary theory and a tool for discovery. *EMBO Rep* 20:e46992.

McFaline-Figueroa JR, Vevea J, Swayne TC, Zhou C, Liu C, Leung G, Boldogh IR, Pon LA. 2011. Mitochondrial quality control during inheritance is associated with lifespan and mother-daughter age asymmetry in budding yeast. *Aging Cell* 10:885–895.

Michel AH, Kornmann B, Dubrana K, Shore D. 2005. Spontaneous rDNA copy number variation modulates Sir2 levels and epigenetic gene silencing. *Genes Dev* 19:1199–1210.

Odani T, Shimma Y, Wang X-H, Jigami Y. 1997. Mannosylphosphate transfer to cell wall mannan is regulated by the transcriptional level of the MNN4 gene in *Saccharomyces cerevisiae*. *FEBS Lett* 420:186–190.

Ouspenski II, Elledge SJ, Brinkley BR. 1999. New yeast genes important for chromosome integrity and segregation identified by dosage effects on genome stability. *Nucleic Acids Res* 27:3001–3008.

Park PU, Defossez PA, Guarente L. 1999. Effects of mutations in DNA repair genes on formation of ribosomal DNA circles and life span in *Saccharomyces cerevisiae*. *Mol Cell Biol* 19:3848–3856.

Pernice WM, Swayne TC, Boldogh IR, Pon LA. 2018. Mitochondrial tethers and their impact on lifespan in budding yeast. *Front Cell Dev Biol* 5.

Peter J, de Chiara M, Friedrich A, Yue J-X, Pflieger D, Bergström A, Sigwalt A, Barre B, Freel K, Llored A, et al. 2018. Genome evolution across 1,011 *Saccharomyces cerevisiae* isolates. *Nature* 556:339–344.

Ping HA, Kraft LM, Chen W, Nilles AE, Lackner LL. 2016. Num1 anchors mitochondria to the plasma membrane via two domains with different lipid binding specificities. *J Cell Biol* 213:513–524.

Salim D, Bradford WD, Freeland A, Cady G, Wang J, Pruitt SC, Gerton JL. 2017. DNA replication stress restricts ribosomal DNA copy number. *PLoS Genet* 13:e1007006.

Sampaio-Marques B, Felgueiras C, Silva A, Rodrigues M, Tenreiro S, Franssens V, Reichert AS, Outeiro TF, Winderickx J, Ludovico P. 2012. SNCA (α-synuclein)-induced toxicity in yeast cells is dependent on Sir2-mediated mitophagy. *Autophagy* 8:1494–1509.

Sinclair DA, Mills K, Guarente L. 1997. Accelerated aging and nucleolar fragmentation in yeast sgs1 mutants. *Science* 277:1313–1316.

Spinazzi M, Casarin A, Pertegato V, Salviati L, Angelini C. 2012. Assessment of mitochondrial respiratory chain enzymatic activities on tissues and cultured cells. *Nat Protoc* 7:1235–1246.

Swygert SG, Senapati S, Bolukbasi MF, Wolfe SA, Lindsay S, Peterson CL. 2018. SIR proteins create compact heterochromatin fibers. *Proc Natl Acad Sci USA* 115:12447–12452.

Torres EM, Sokolsky T, Tucker CM, Chan LY, Boselli M, Dunham MJ, Amon A. 2007. Effects of aneuploidy on cellular physiology and cell division in haploid yeast. *Science* 317:916–924.
